# Development and content validity testing of patient-reported outcome (PRO) items to assess chest congestion associated with the common cold for use in children and adolescents

**DOI:** 10.1186/s41687-022-00465-8

**Published:** 2022-05-28

**Authors:** Rob Arbuckle, Chris Marshall, Laura Grant, Kate Burrows, Helmut H. Albrecht, Tim Shea

**Affiliations:** 1 Adelphi Values Patient-Centered Outcomes, Bollington, UK; 2 Adelphi Values Patient-Centered Outcomes (at the time research was conducted), Bollington, UK; 3 Herbert Wertheim College of Medicine, Cellular Biology and Pharmacology, Florida International University, Miami, FL USA; 4 RB Health (US), LLC, Parsippany, New Jersey, USA

**Keywords:** Patient-reported outcome development, Pediatrics, Qualitative research, Concept elicitation, Cognitive debriefing

## Abstract

**Background:**

This article describes qualitative interviews conducted with children (aged 6–11), adolescents (aged 12–17), and adults with the common cold as well as parents/caregivers of the 6–8-year-old children. The aim was to support the refinement and content validity testing of patient-reported outcome (PRO) items assessing chest congestion that could be used as pediatric clinical trial endpoints. Feasibility and acceptability of administering the PRO items electronically on a hand-held touch-screen device were also evaluated. The sample included children aged 6–8 years (n = 14), 9–11 years (n = 13), adolescents aged 12–17 years (n = 12), and adults (n = 10), all of who had current (n = 38) or recent (n = 11) cold. Both concept elicitation (CE) and cognitive debriefing (CD) interviews were conducted with all of these participants, conducted over in two rounds. Ten parents/caregivers of participants aged 6–8 years were also interviewed (separately from their child) regarding how they thought their children would understand the items. The CE interviews explored the qualitative experience of having chest congestion and related symptoms of the common cold. Following their CE interview, participants completed draft items on an electronic patient-reported outcome (ePRO) device twice daily for 2–5 days prior to their CD interview. During the CD interview participants were asked about relevance, understanding and interpretation of the draft PRO items. Qualitative analysis of the interview data and descriptive analyses of the ePRO data were conducted following both rounds of interviews, with modifications to the items implemented following Round 1 and tested in Round 2.

**Results:**

Eight symptoms were reported by children during concept elicitation. Findings from the child, adolescent, and adult/parent interviews supported revisions to the items and enabled the selection of the best performing items. The results provided evidence that the final items were well understood by participants and relevant to their experiences of chest congestion as part of a common cold. Findings also provide support for using the same items across age groups.

**Conclusions:**

The results of the CE and CD interviews provide evidence supporting the content validity of new PRO items assessing the experience of chest congestion symptoms associated with common cold experienced by children, adolescents, and adults.

**Supplementary Information:**

The online version contains supplementary material available at 10.1186/s41687-022-00465-8.

## Background

The common cold, an upper respiratory tract infection (URTI), is the most frequently occurring acute illness experienced in pediatric and adult populations and for which approximately 25 million individuals seek medical attention per year in the USA [1]. The etiologic agents behind the cold are more than 200 virus species, but most commonly rhinovirus [2]. Symptoms of the common cold include fever, cough, chest congestion, nasal congestion, sore throat, headache, and myalgias, with the most common symptoms experienced being sore throat (50%) and cough (40%) [3, 4]. These symptoms can lead to activity, functioning and participation limitations and thus have an adverse effect on health-related quality of life (HRQoL) [4–6]. While symptomology experienced across children and adults are largely the same, children experience colds more often than adults, up to ten times per annum, accounting for 22 million missed days of school [2, 5].

Over the counter (OTC) treatments represent a major method of active management of the common cold. However, there is limited evidence of efficacy, specifically in children, that is based on controlled clinical trials [1]. Currently, Guaifenesin, is the only Food and Drug Administration (FDA)-approved expectorant ingredient used in non-prescription (OTC) cold medications in the USA, indicated to treat chest congestion and cough caused by the common cold in adults and children aged four years and over. The labelled indication for Guaifenesin states that it “helps loosen mucus and thin bronchial secretions to rid the bronchial passageways of bothersome mucus and make coughs more productive” [7].

There is both a paucity of PRO measures specific to the common cold and limited evidence from controlled clinical studies for the efficacy of OTC medicines developed to target chest congestion in a pediatric population. Moreover, valid and reliable PRO measures are arguably essential for evaluating severity in a condition defined by symptoms experienced by the patient such as chest congestion. The research described in this paper builds upon previous work conducted to develop and psychometrically validate a PRO measure—the Child Cold Symptom Questionnaire (CCSQ)—to assess the most important and burdensome cold symptoms in children aged 6–11 years [8, 9]. During the development of the CCSQ it was recognised that chest congestion is a particularly difficult symptom to assess accurately in pediatric populations. The present research used as a starting point selected items from the CCSQ that assessed the symptom of chest congestion. In addition, children’s, adolescents’, and adults’ experiences and descriptions of chest congestion were further explored through additional prospective qualitative research, which included both concept elicitation and cognitive debriefing of a large set of items. Thus, this research has involved in-depth qualitative interviews to support the development and content validity testing of a larger pool of items, all focused on chest congestion.

Robust, well-established methodologies exist for the development of PROs in adults as summarised by the US Food and Drug Administration (FDA) [10]. These methods can be applied to pediatric work but with additional considerations not encountered in adults [8, 9, 11–13]. For example, wide variation exists in linguistic, cognitive, and motor capacities among children of the same age. Electronic clinical outcome assessment (eCOA) offers benefits over traditional paper collection of clinical outcome assessment data and empowers patient populations with cognitive limitations [14, 15]. eCOA is therefore the preferred method of data capture due to high patient adherence and superior data quality, particularly for a diary that will be completed daily or more frequently [16]. The feasibility and acceptability of administering the PRO items electronically on a hand-held touch-screen device were also evaluated. Some of the PRO items relevant to this study had already been developed in previous research [8]; the items were tested and refined in the cognitive debriefing interviews within this study.

## Methods

### Aims of the study

The primary objective of this qualitative research was to develop and conduct content validity testing of a draft electronic PRO (ePRO) instrument designed to measure chest congestion symptoms experienced during a common cold by children, adolescents, and adults. A secondary goal was to qualitatively examine the extent to which the experience of chest congestion is similar or different between child, adolescent, and adult populations.

### Sample and recruitment

This was a qualitative interview study, with interviews conducted across two rounds (n = 25 interviews in each round). A total of 49 participants with current (n = 38) or recent colds (n = 11) completed all study activities. The sample included children aged 6–8 years (n = 14), 9–11 years (n = 13), adolescents aged 12–17 years (n = 12), and adults (n = 10). It was planned that the majority of participants should be experiencing a cold at the time of the study, however, some participants who had recently (in the 2 weeks preceding enrolment) experienced a cold were also targeted for inclusion to ensure the instrument is acceptable and has content validity in individuals who have recently recovered from a cold, as well as those in the acute phase. Ten parents/caregivers of participants aged 6–8 years were also interviewed (separately from their child) to obtain their feedback on their child’s ability to read and understand the items and the feasibility of incorporating daily diary completion into their daily routine.

Participants were recruited through advertisements by a specialist patient recruitment agency in two geographical locations in the USA: Boston, Massachusetts and Chicago, Illinois. Sampling quotas were established to ensure recruitment of participants with a range of demographic and clinical characteristics. The recruitment agency completed screening with each patient (for adults) or parent/guardian to confirm eligibility and written informed consent was obtained from the adults, from the parent/guardian (for children and adolescents) and assent from the children and adolescents, prior to any other study activities.

For inclusion in the study, all participants had to be at least 6 years old, a native US-English speaker, willing and able to provide written assent and willing and able to participate in two separate interviews and to take the ePRO device home to complete the diary for days 2–5 between the interviews. The children also had to be of typical or higher reading level for their age based on parent/caregiver report. An additional inclusion criterion for participants currently experiencing cold symptoms was having a common cold or URTI (in the opinion of the participant [for adults] or their parent/guardian [for children/adolescents]) but being otherwise healthy. During screening the participants had to complete the Child Cold Symptom Checklist which includes 3 questions which ask about severity of chest congestion symptoms (how hard it was to breathe air deep into their chest, how much their chest felt ‘full of mucus [the goo that comes out of your nose]’ and how hard it was to clear their chest) with a recall period of ‘today’. The participants currently experiencing cold symptoms had to choose a response of at least “a little” or “a little hard” on the five-point response scales (not at all, a tiny bit, a little/a little hard, some/hard, a lot/very hard) associated with at least one of those three chest congestion screening items. Those participants also had to have a response of at least ‘bad’ to the Child Global question, ‘How bad is your cold today?’ For participants who had recently experienced cold symptoms, an additional inclusion criterion was the experience of a common cold in the 2 weeks preceding enrolment in the study, but otherwise healthy. Those participants also had to have experienced chest congestion, cough, and at least one other cold symptom in the 2 weeks prior to enrolment. Diagnosis of the common cold was not confirmed by a clinician because patients rarely consult with a clinician for the common cold and there is no definitive test to confirm diagnosis.

The parents/caregivers who participated had to be the parent/caregiver of a child with current cold symptoms who met all of the relevant study selection criteria and to be one of the child’s primary caregivers at least 50% of the time. They were also required to be literate native US-English speakers.

Participants were excluded if cold or flu symptoms had been experienced continuously for more than 2 weeks as this suggested a cause other than the common cold. They were also excluded if experiencing severe symptoms, including a high fever (above 101.5F) that could limit their ability to participate comfortably. Additionally, participants were excluded if currently receiving antibiotics or other prescribed medicine or had received a diagnosis of sinusitis, otitis media, tonsillitis, strep throat, laryngitis, pertussis, or pneumonia. Allergies, psychiatric or cognitive conditions (including an uncontrolled psychiatric condition that may affect their ability to participate in interview), history of drug, alcohol or tobacco use were also exclusion criteria.

### Interview procedure

**Fig. 1:**
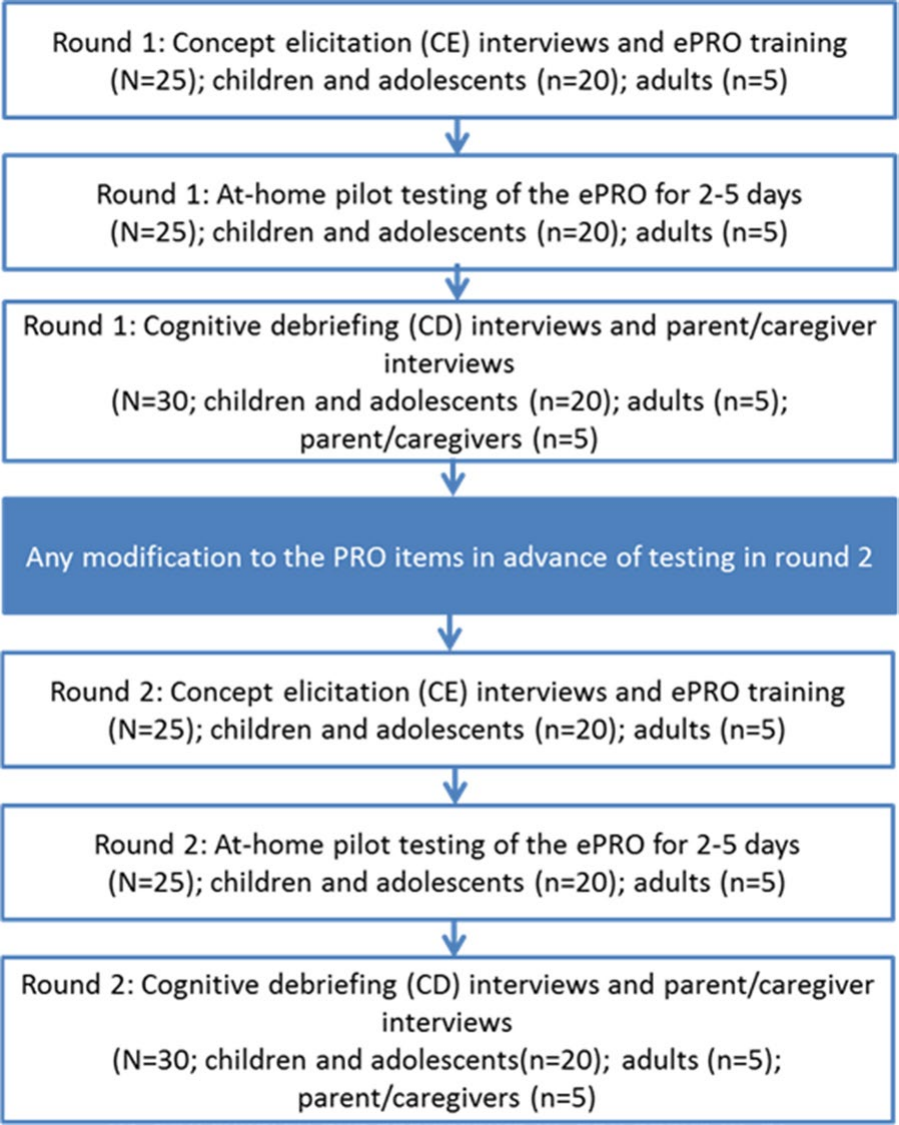
Overview of study methodology

Semi-structured, face-to-face, qualitative interviews were conducted, and all interviews were conducted by experienced interviewers trained in pediatric qualitative interviewing. The interview procedure involved three stages: visit 1 concept elicitation (CE) interview (45 min), at home ePRO completion stage, and visit 2 cognitive debriefing (CD) interview (45 min). An overview is provided in Fig. 1. At visit 1, discussion started with open-ended, exploratory questions encouraging participants to spontaneously talk about their experience of chest congestion and other cold-related symptoms [17]. Probes or direct questions were used to help participants to expand further. For some of those who were less forthcoming (children especially), they were asked to draw a picture of what their chest felt like during their cold and then talk about the picture. At the end of the interview, participants were trained on how to complete the ePRO diary. For the younger children, the parents/caregivers also observed the training session so that they could help their child if necessary.

Participants were given the ePRO diary device to take home and instructed to complete the items twice daily (morning and afternoon) for 2–5 days. Participants returned to take part in the CD interview to assess understanding and relevance of the ePRO diary items, recall period, response scale, and instructions, as well as the usability of the ePRO diary. The ‘think aloud’ method used involved participants speaking aloud their thoughts as they read each instruction and completed each item. This approach was supplemented by detailed cognitive interviewing questions to confirm relevance and understanding.

### Selection/development and refinement of PRO items

Items were initially selected/developed to assess the following seven concepts associated with chest congestion, based on the findings of previous research in the common cold and with input from a clinical expert [8]:

- Difficulty breathing
- Chest tightness
- Chest pain
- Chest feels heavy
- Chest feels full of mucus/stuffed up/clogged up
- Difficulty clearing mucus
- Wheeze/noise when breathing

**Fig. 2:**
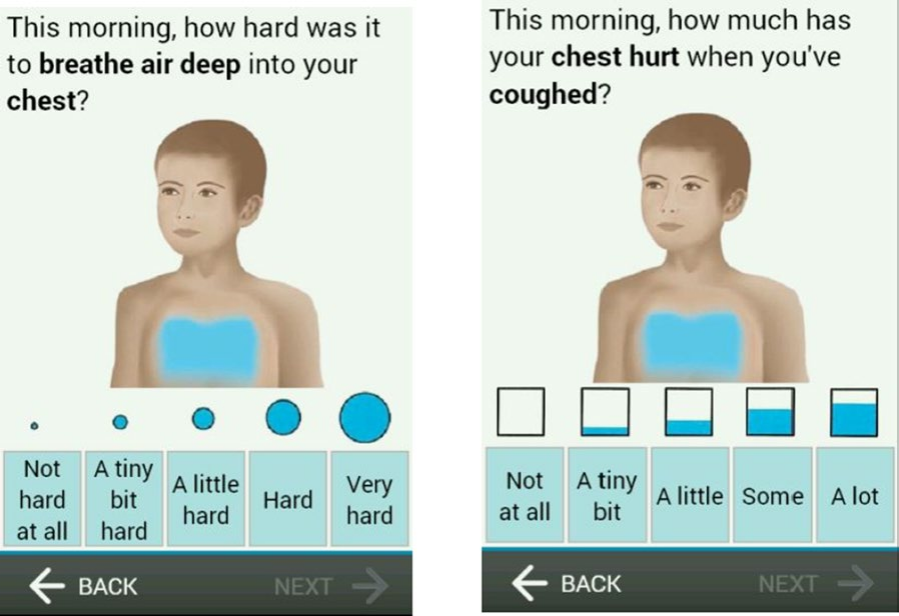
Example of item screen shots showing the two different visual response scales

For each concept, at least two item versions were tested, to explore which wording would be best understood. For each concept, one version was included on the ePRO diary and completed by the participants at home. Alternative versions (in some cases, several alternative versions) were then only presented to the participants on paper during the CD interview. A summary of all items debriefed, changes made between rounds and rationales for those changes is provided in Table 1. In addition, two different pictorial response scales were tested with verbal descriptors for each response option—one using circles of increasing size to indicate severity and one using boxes that become gradually filled to indicate frequency (see examples in Fig. 2).

**Table 1:** Item tracking matrix summarising the item versions tested in each round of cognitive debriefing and the final items taken forward for psychometric validation

Concept	Item tested in round 1	Item tested in round 2	Rationale	Final item
Difficulty breathing	ePRO1. “…how hard was it to breathe air deep into your chest?”	UNCHANGED: ePRO1. “…how hard was it to breathe air deep into your chest?”	Well understood and equally relevant to Paper 1 but has fewer words for children	RETAIN ePRO1. “…how hard was it to breathe air deep into your chest?”
	Paper1. “…how hard was it to breathe air deep into your chest because of your cold?”	UNCHANGED: Paper1. “…how hard was it to breathe air deep into your chest because of your cold?”	ePRO1 performed similarly and has fewer words for children	DELETE
Chest tightness	ePRO2. “…how tight did your chest feel because of your cold?”	UNCHANGED: ePRO2. “…how tight did your chest feel because of your cold?”	Paper2 performed similarly and has fewer words for children	DELETE
	Paper2. “…how tight did your chest feel?”	UNCHANGED: Paper2. “…how tight did your chest feel?”	Well understood and equally relevant to Paper1, but has fewer words for children	RETAIN and move to ePRO ePRO2. “…how tight did your chest feel?”
Chest pain	ePRO3. “…how much has your chest hurt when you’ve coughed?”	UNCHANGED: ePRO3. “…how much has your chest hurt when you’ve coughed?”	Was well understood and relevant to the majority of participants	RETAIN ePRO3. “…how much has your chest hurt when you’ve coughed?”
	Paper3. “…how much has your chest hurt due to being stuffed up?”	UNCHANGED: Paper3. “…how much has your chest hurt due to being stuffed up?”	Poorly understood item across the different age groups	DELETE
Chest feels heavy	ePRO4. “…how heavy did your chest feel?”	UNCHANGED: ePRO4. “…how heavy did your chest feel?”	Both items assessing chest heaviness performed similarly yet ePRO4 followed the same format as the chest tightness item and so it was agreed that children would find this easier to understand	RETAIN ePRO4. “…how heavy did your chest feel?”
	Paper4. “…how much of the time has your chest felt heavy?”	UNCHANGED: Paper4. “…how much of the time has your chest felt heavy?”	ePRO4 was easier for the younger participants to read and understand	DELETE
Chest feels full	ePRO5. “…how much did your chest feel full of mucus (the goo that comes out of your nose)?”	AMENDED WORDING: ePRO5. “…how much did your chest feel full of mucus (goo)?”	Item performed equally well with the term ‘goo’ and ‘goo that comes out of your nose’ and so it was agreed that fewer words would be easier for children to understand	RETAIN WITH WORDING FROM ROUND TWO ePRO5. “…how much did your chest feel full of mucus (goo)?”
	ePRO6. “…how stuffed up did your chest feel?”	UNCHANGED: ePRO6. “…how stuffed up did your chest feel?”	Performed similarly to Paper5a but has fewer words for children	RETAIN ePRO6. “…how stuffed up did your chest feel?”
	Paper5a. “…how much did you feel stuffed up in your chest?”	UNCHANGED: Paper5a. “…how much did you feel stuffed up in your chest?”	ePRO6 was better understood and easier for younger participants to understand	DELETE
	Paper5b. “…how much did you feel clogged up in your chest?”	MOVED TO ePRO: ePRO5b. “…how much did you feel clogged up in your chest?”	ePRO5c was better understood and easier to read for the younger participants	DELETE
	Paper 5c. “…how clogged up did your chest feel?”	MOVED TO ePRO: ePRO5c. “…how clogged up did your chest feel?”	Performed similarly to Paper 5b but was simpler and shorter for children to read	RETAIN (may require further testing) ePRO5c. “…how clogged up did your chest feel?”
		NEW ITEM: ePRO5d. “…how full of stuff did your chest feel?”	Misunderstood by almost a third of participants	DELETE
	Paper6c. “…how clear did your chest feel?”	UNCHANGED: Paper6c. “…how clear did your chest feel?”	This was the only item in which the response scale was reversed and so it was agreed that it should be removed to avoid confusion	DELETE
Difficulty clearing mucus	ePRO7. “…how hard was it to clear your chest?”	UNCHANGED: ePRO7. “…how hard was it to clear your chest?”	‘Difficulty clearing mucus’ is a key symptom of chest congestion and so it was agreed that this item should be retained to fully assess this concept	RETAIN (may require upfront question) ePRO7. “…how hard was it to clear your chest?”
	Paper6a. “…how hard was it to clear your throat?”	UNCHANGED: Paper6a. “…how hard was it to clear your throat?”	‘Difficulty clearing mucus’ is a key symptom of chest congestion and so it was agreed that this item should be retained to fully assess this concept	RETAIN and move to ePRO (may require upfront question) ePRO6a. “…how hard was it to clear your throat?”
	Paper6b. “…how hard was it to blow your nose?”	UNCHANGED: Paper6b. “…how hard was it to blow your nose?”	This item was not considered adequately relevant to chest congestion and could be confusing in combination with the added instruction to focus on chest rather than nose	DELETE
	Paper8b. “…how hard was it to cough up mucus (goo) from your chest?”	MOVED TO ePRO AND AMENDED WORDING: ePRO8b. “…how hard was it to cough up mucus (gunk) from your chest?”	The term ‘goo’ was better understood and more relevant than the term ‘gunk’	RETAIN WITH ORIGINAL WORDING (may require question upfront) ePRO8b. “…how hard was it to cough up mucus (goo) from your chest?”
Noise when breathing	ePRO8. “…how much did you wheeze (make a noise) when you breathed?”	ePRO8. “…how much did you wheeze (make a noise) when you breathed?”	Noise when breathing was not a concept of interest and this item had poor relevance	DELETE
	Paper7a. “…how much did your chest make a rattling noise when you breathed?”	Paper7a. “…how much did your chest make a rattling noise when you breathed?”	Noise when breathing was not a concept of interest and this item had poor relevance and understanding	DELETE
	Paper7b. “…how much have you noticed a sharp noise when you breathed in or out?”	Paper7b. “…how much have you noticed a sharp noise when you breathed in or out?”	Noise when breathing was not a concept of interest and this item had poor relevance and understanding	DELETE

Interviews were conducted in two rounds to allow opportunity to make changes to the draft PRO items based on interim findings from Round 1 and then test those changes in Round 2.

### Qualitative analysis

All interviews were audio-recorded and transcribed verbatim. Qualitative analysis of interview transcripts involved thematic analysis methods and Atlas.Ti software [18] with quotes sorted and grouped by conceptual domain. It was also coded whether concepts were reported spontaneously or were probed. Analysis of the CD interviews assessed the relevance and understanding of each item, instruction, response scale, and recall period. Each participant was assigned a unique participant identification number based on their demographics to aid sub-group analysis, while preserving anonymity. The first four digits form a unique participant number. This is followed by either CC for Current Cold or RC for Recent Cold. Next is F for female or M for male. The final two digits indicate the participants’ age. Where a quote was from a parent a P was also added to the end. For example, a quote coded 0104-RC-M-13 was from a 13 year-old male with a recent cold.

## Results

### Demographics

Forty-nine participants with current or recent colds participated; 38 participants had a current cold (and were enrolled less than 72 h after cold onset) and 11 participants had a recent cold (within the past two weeks). There were similar numbers in each of the pediatric age groups: 6–8 years (n = 14), 9–11 years (n = 13) and 12–17 years (n = 12) and an almost even gender divide (26 males and 23 females). The sample provided a good representation of race and ethnicity. Geographical diversity was also achieved: a total of 31 participants were recruited from Chicago and 18 from Boston. Full demographics are provided in Table 2. Ten parents/caregivers of 6–8 year-old children were interviewed. The parent/caregiver sample had a mean age of 39.6 years and all were parents (i.e. either mother or father) of the child. Although the majority of parents were female (n = 8, 80%), the pre-specified quota of recruiting at least two male parents was met.

**Table 2:** Demographic characteristics of the total sample

Description		Current cold (n = 38)	Recent cold (n = 11)	Total sample (n = 49)
*Age*				
Mean	Total sample	21.7	20.2	19.1
	Child	8.7	8.0	8.5
	Adolescent	14.1	14.7	14.3
	Adult	53.5	–	53.5
Range	Total sample	6–74	6–74	6–74
	Child	6–11	6–11	6–11
	Adolescent	12–16	13–17	12–17
	Adult	26–74	–	26–74
*Gender, n (%)*				
Male	Total sample	21 (55.3)	5	26
	Child	10	3	13
	Adolescent	6	2	8
	Adult	5	–	5
Female	Total sample	17 (44.7)	6	23
	Child	9	5	14
	Adolescent	3	1	4
	Adult	5	–	5
*Ethnicity, n (%)*				
Hispanic or Latino	Total sample	8 (21.1)	1 (9.1)	9 (18.4)
	Child	4	1	5
	Adolescent	2	–	2
	Adult	2	–	2
Non-Hispanic/Latino	Total sample	30 (78.9)	10 (90.9)	40 (81.6)
	Child	15	7	22
	Adolescent	7	3	10
	Adult	8	–	8
*Race, n (%)*				
White	Total sample	25 (65.8)	10 (90.9)	35 (71.4)
	Child	12	7	19
	Adolescent	7	3	10
	Adult	6	0	6
Black/African American	Total sample	5 (13.2)	0 (0.0)	5 (10.2)
	Child	3	0	3
	Adolescent	–	0	0
	Adult	2	0	2
Multi-racial	Total sample	1 (2.6)	0 (0.0)	1 (2.0)
	Child	1	0	1
	Adolescent	0	0	0
	Adult	0	0	0
Other—Hispanic* *This was written in by the respondents, hence the overlap with ethnicity	Total sample	4 (10.5)	0 (0.0)	4 (8.16)
	Child	2	0	2
	Adolescent	2	0	2
	Adult	0	0	0
Other—not specified	Total sample	1 (2.6)	0 (0.0)	1 (2.0)
	Child	0	0	0
	Adolescent	0	0	0
	Adult	1	0	1
Missing data	Total sample	2 (5.3)	1 (9.1)	3 (6.12)
	Child	2	1	3
	Adolescent	0	0	0
	Adult	0	0	0

### Concept elicitation results

**Fig. 3:**
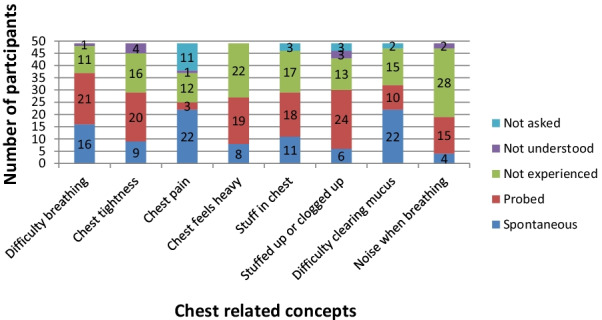
Summary of concept elicitation results

An overview of the concepts reported throughout the CE interviews is provided in Fig. 3. Chest-related concepts reported included: difficulty breathing; chest tightness; chest pain; the chest feeling heavy, stuffed up or clogged up; difficulty clearing mucus, and noises when breathing. Overall, difficulty breathing was the most frequently reported concept. Difficulty clearing mucus and chest pain were reported spontaneously most frequently. Noise when breathing was the least frequently reported. Aside from noise when breathing, the remainder of the concepts were reported by just over half of the sample (substantially more for the difficulty breathing). The concepts reported spontaneously most often were difficulty clearing mucus and chest pain. Detailed findings for the most frequently reported concepts are provided below. The findings from the interviews with parent/caregivers are not reported here, but generally corroborated their child’s interview findings, with some additional details provided occassionally.

#### Difficulty breathing

A total of 37 (37/49, 75.5%) participants reported difficulty breathing; 16 participants (16/49, 32.7%) spontaneously talked about difficulty breathing as a symptom of their cold, while a further 21 participants (21/49, 42.9%) reported difficulty breathing only when asked directly. There did not appear to be observable differences between age groups, with all age groups spontaneously reporting difficulties with breathing (n = 16). Descriptions that participants used to talk about difficulties breathing included: being unable to breathe properly and heavier/harder breathing, *“I couldn’t breathe that way, but it was like the air was just harder and harder to get up and out” (0211-RC-M-17)*.

#### Chest tightness

Twenty-nine participants (29/49, 59.2%) reported experiencing chest tightness; nine spontaneously. One six-year-old participant (0131-CC-M-6) described chest tightness as *‘hurting chest’* suggesting he may not have been able to distinguish chest tightness from chest pain. However, it is equally possible the chest tightness was simply a painful sensation for that child. While it was reported reasonably frequently, relatively few children/adolescents spontaneously reported chest tightness (n = 6/39), perhaps indicating it is a challenging concept for children/adolescents to comprehend and articulate. Only one child in the 6–8 years age group referred to his chest feeling ‘tight’ spontaneously when describing his clogged-up chest, *‘like my chest like is really tight’* (0206-CC-M-7). However, due to the generally small number of spontaneous reports of chest tightness, the difference between age groups results needs to be interpreted with caution.

#### Chest pain

Twenty-five participants (25/49, 51.0%) reported experiencing chest pain (22 spontaneously). Of note, specific chest pain questions were not asked directly in the Round 1 interviews. Despite this, chest pain was still reported spontaneously by 11/26 (42.3%) participants. As a result, the concept of chest pain was added to the interview guide and participants in Round 2 were asked directly about their experiences of chest pain if they did not spontaneously report this concept. Participants discussed chest pain in several different ways but almost half of the sample used the term ‘hurts’. Of the 22 participants who reported that different symptoms could be the cause of chest pain, 50% (n = 11) reported chest pain was caused by coughing, five mentioned breathing problems and two chest congestion.

#### Other symptoms

Thirty-two participants (32/49, 65.3%) reported difficulty clearing mucus from their chest; 22 spontaneously. Thirty participants (30/49, 61.2%) reported experiencing feeling ‘stuffed up or clogged up in their chest’; 6 participants spontaneously. Twenty-nine participants (29/49, 59.2%) reported getting ‘stuff’ in their chest; 11 spontaneously. Twenty-seven participants (27/49, 55.1%) reported experiencing chest heaviness (8/49, 16.3% spontaneously; 19/49, 41.3% only when asked directly). Nineteen participants (19/49, 38.8%) reported noise when breathing with their cold; four spontaneously. There were no clear differences between age groups.

### Cognitive debriefing results

**Fig. 4:**
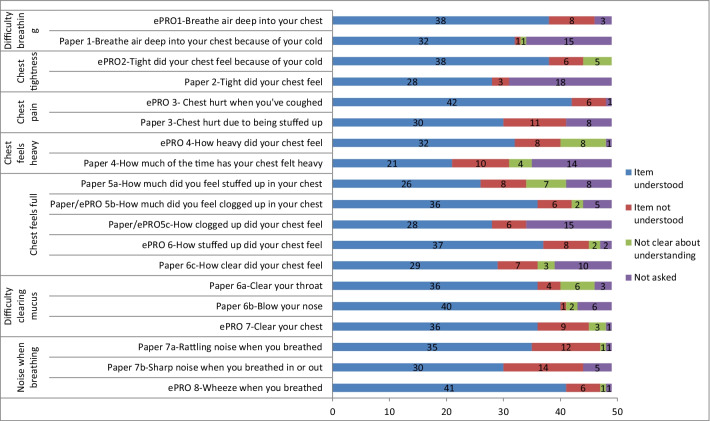
Summary of cognitive debriefing results: understanding

**Fig. 5:**
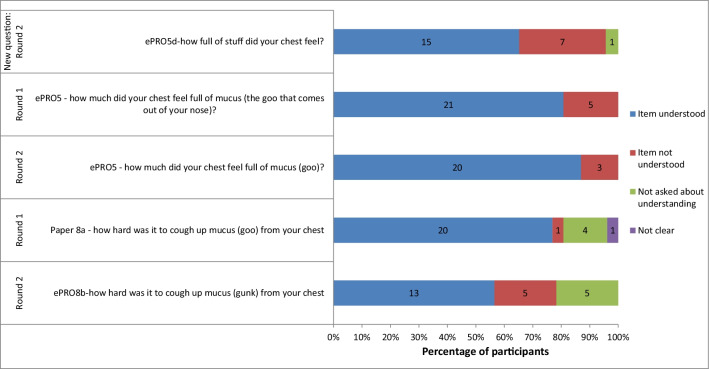
Summary of cognitive debriefing results: understanding of additional items

A summary of understanding for the items tested during the CD interviews is presented in Figs. 4 and 5. Due to different numbers of participants being debriefed on each item, comparing the percentage of participants asked who did not understand each item is the most useful way to interpret understanding. Almost all of the items were well understood across a large majority of the sample. The only items misunderstood by more than 10 participants (approximately 20%) were: Paper3 ‘chest hurt due to being stuffed up’ (n = 11), Paper7a ‘rattling noise’ (n = 12), and Paper7b ‘sharp noise’ (n = 14). All of these item versions were ultimately deleted based on the CD results.

Item Paper7b assessing ‘a sharp noise when breathing’ was the item most frequently misunderstood by participants. An item added after the first round of interviews (ePRO5d ‘how full of stuff did your chest feel’) was misunderstood by almost a third of the participants and more participants understood the term ‘goo’ than the term ‘gunk’ debriefed on it (7/23, 30.4%). As a result this item was deleted.

**Fig. 6:**
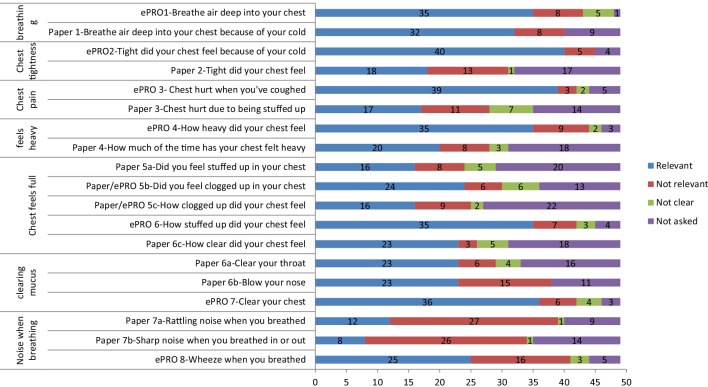
Summary of cognitive debriefing results: relevance

**Fig. 7:**
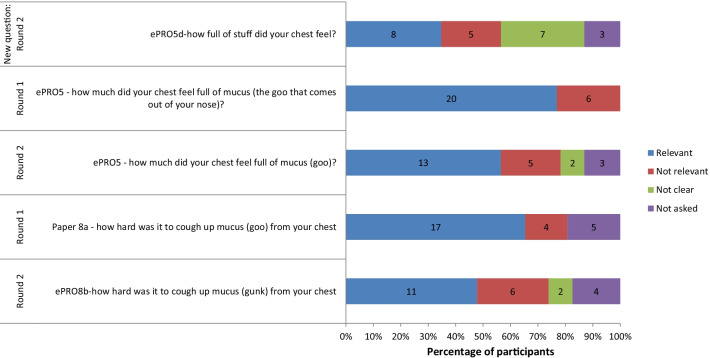
Summary of cognitive debriefing results: relevance of additional items

A summary of the relevance associated with each item is presented in Figs. 6 and 7. Item Paper7a assessing a rattling noise was the least relevant item followed closely by item Paper7b assessing a sharp noise. Both were deleted. It was difficult to interpret the relevance of the newly added item (ePRO5d ‘how full of stuff did your chest feel’) as relevance was unclear for 7/23 participants debriefed on that item. It was decided to delete all ‘noises when breathing’ items as they were the least relevant. All items assessing ‘difficulty breathing’, ‘chest feeling heavy’ and ‘chest feeling full’ seemed to have strong relevance, with less than 10/49 suggesting they were not relevant. For the ‘difficulty clearing mucus’ items, only the ‘blow your nose’ item had problematic relevance and was deleted. Detailed CD results for each of the instructions and items, including both understanding and relevance are provided in Additional file 1: Detailed Cognitive Debriefing Results.

Participants were specifically asked to provide feedback on the two options for visual aids for the response options (e.g. Fig. 2: increasingly filled boxes and circles of increasing sizes). Details are again provided in Additional file 1: Detailed Cognitive Debriefing Results. Overall, participants found both types of response scale easy to understand. Given that both the increasingly filled boxes and circles of increasing sizes performed well and were acceptable, both visual aids were retained on different items for inclusion in the validation study.

The recall periods were also well understood and the majority of participants had no difficulty understanding or using the recall periods specified. There did not appear to be any observable differences between the age groups in terms of participants who reported difficulties recalling over the specified time periods: three in the 6–8 year-old age group, four in the 9–11 year-old age group, five in the 12–17 year-old age group, and the remaining three in the adult age category.

### Revisions made to the items based on the findings

The item tracking matrix in Table 1 provides full details of the item versions tested in each round, the rationale for modifications, and the final item versions taken forward into psychometric validation. In total, six modifications were made to the ePRO diary and items following Round 1. These amendments included inclusion of an instruction screen, modification to items ePRO5 and Paper 8, implementing Paper 5b and 5c items on the ePRO device, and adding a new item including the term ‘full’. Following Round 1, an instruction screen was added to remind participants to focus on chest rather than nasal symptoms to avoid risk of confusion. All 23 participants in Round 2 confirmed that they understood this instruction as intended. Following both rounds of interviews, for all items it was decided not to include the attribution ‘because of your cold’, to reduce the number of words in the items and make them as simple as possible for the children to read.

### Usability and feasibility results

The morning diary took participants an average of 1 min 43 s to complete; the evening diary, 2 min 36 s. Thus, the majority were completing the diary in less than 3 min, providing evidence that completing the diaries was not burdensome. All 49 participants stated that the time taken to complete the ePRO items was acceptable and 9/10 parent/caregivers indicated it was not an issue and easy to fit into the daily routine. Additionally, 27 out of 32 participants (84.4%) reported they always completed the ePRO diary at the correct times. Participant feedback on the experience of completing the diary was very positive. A list of features of the ePRO diary/experience liked by participants is presented in Table 3. All participants across all age groups found the ePRO device easy to use and were happy with the format. Only minor problems were identified with the device with 100% of participants reporting overall that the device was generally ‘easy to use’. The main changes suggested by participants related to being able to adjust the device alarm volume and alarm window.

**Table 3:** Features of the ePRO diary and/or at-home completion phase that participants liked

Likes	Quotes
Simple and easy to use (n = 15)	*“Um the questions were very um,—they were very simple—very easy, very understandable.”* (0106-CC-F-53)
Small and portable (n = 7)	*“Well, uh, first of all, I could take it with me. It was a short—a small little—like a little cell phone.”* (0103-CC-F-70)
Similar questions (n = 5)	*“Um, I liked how it like—how it would do the same exact questions, um, each day and like so there was no like different order, and I would know like what that one actually meant after like a period of time.”* (0210-CC-F-14)
Short (n = 4)	*“And the thing I liked about it, um, when you do it—it’s not really that long. It’s just a little short, like five or six pages—and that’s it.”* (0101-CC-F-8)
Responsibility (n = 3)	*“Um, what I liked about the device is that, um, I had my, uh—I had a big responsibility and I had to take care of it and—and, uh—and it just gave me a nervous feeling when I didn’t answer it.”* (0119-CC-M-11)
Quick (n = 3)	*“Well, it was like—it wasn’t hard clicking them. Like it was fast.”* (0121-CC-M-12)
Response options (n = 3)	*“The options that they gave were really—um, wide enough for you to really answer precisely.”* (0108-CC-M-51)
Personalised (n = 3)	*“Uh, I liked that it was personalised for me—it made me feel kind of important about taking care of myself.”* (0108-CC-M-51)
Prefer phone to paper (n = 3)	*“Like that it was in a phone and not in—not in a piece of paper.”* (0111-CC-M-12)
Flow between items (n = 2)	*“I liked the fact that everything was next. You had to roll into everything next—it was not necessary to have to go back.”* (0106-CC-F-53)
Illustrations (n = 2)	*“I could see how they could—show the picture, you know, kind of, too was helpful—to figure out what they were asking.”* (0104-CC-F-50)
Alarms (n = 2)	*“What did you like about it?” “And the alarm was really helpful.”* (0209-CC-F-16)
Fun (n = 2)	*“It was fun—because um, I got to answer like, questions and I got to go on the device like, every morning and afternoon—it made me fun like answering all these questions.”* (0126-CC-F-7)

## Discussion

This qualitative study developed and evaluated a new ePRO assessment of chest symptoms associated with the cold in pediatric populations. The research reported here built on previous research [8] focussed on common cold symptoms in children more generally. The items developed and tested provide additional options to researchers for the pediatric assessment of chest congestion associated with the common cold in the context of clinical trials, conducted to evaluate the efficacy of cough and cold medication in children. An in-depth approach was taken that included interviews with children, adolescents, parent/caregivers of children and adults. A rich array of chest-related concepts were identified from the CE part of the interviews, including: difficulty breathing; chest tightness; chest pain; chest heaviness; stuff in chest; stuffed up or clogged up; difficulty clearing mucus and noise when breathing. Anticipating that some children may have difficulty understanding questions related to some of these concepts, for each concept several different versions of items were drafted and tested, with the aim of identifying wording that would be well understood by all. This was successful. The use of pictures associated with each response option also seemed to help the children respond, as has been found elsewhere [11, 12]. The final items were selected based on participant reported understanding and relevance, particularly the level of understanding in the younger participants. The items selected for taking into psychometric validation aim to assess all relevant symptom concepts associated with chest congestion and have the potential to be included in efficacy studies of chest congestion treatments in children, adolescents, or adults with symptoms of the common cold. A final set of ten items were selected for psychometric evaluation covering the following concepts: difficulty breathing, chest tightness, chest pain, chest feels heavy, chest feels full, and difficulty clearing mucus (Table 4).

**Table 4:** Final conceptual framework for items to be included on the ePRO diary for psychometric evaluation

Concept	Item
Difficulty breathing	ePRO1. This morning/this afternoon, how hard was it to breathe air deep into your chest?
Chest tightness	ePRO2. This morning/this afternoon, how tight did your chest feel?
Chest pain	ePRO3. This morning/this afternoon, how much has your chest hurt when you’ve coughed?
Chest feels heavy	ePRO4. This morning/this afternoon, how heavy did your chest feel?
Chest feels full	ePRO5. This morning/this afternoon, how much did your chest feel full of mucus (goo)?
	ePRO6. This morning/this afternoon, how stuffed up did your chest feel?
	ePRO7. This morning/this afternoon, how clogged up did your chest feel?
Difficulty clearing mucus	ePRO8. This morning/this afternoon, how hard was it to clear your chest?
	ePRO9. This morning/this afternoon, how hard was it to clear your throat?
	ePRO10. This morning/this afternoon, how hard was it to cough up mucus (goo) from your chest?

The CD results confirmed that the majority of items were well understood and relevant to participants, with less relevant items removed. The study design included completion of the ePRO items twice daily for 2–5 days prior to the CD interview. This step was valuable to generate evidence that twice daily completion of a number of items was feasible and to give participants experience of reading and answering the items. Collectively, participants across all age groups found the ePRO device easy to use and were happy with the format.

One of the more unusual aspects of this research is that children, adolescents, and adults were interviewed as part of one study and the same items were debriefed with all three age groups. The results provide evidence of a high degree of consistency across the age groups regarding the relevance of the different chest congestion concepts. While there was some evidence of age differences in comprehension, the best performing items were well understood by all. Thus, the findings provide evidence that, if items are worded simply enough, then the same items can be appropriate to use across children, adolescents, and adults.

Study limitations included no clinician-confirmed diagnosis of a cold; however, this reflects the real-world self-management of the common cold. Additionally, the study was only conducted in the USA and further research in other countries would be valuable to confirm the findings reflect the way other cultures describe cold symptoms. In addition to interviewing the children themselves, for the 6–8 year olds children their parents were also interviewed. Proxy reporting in pediatric populations (through parent-report proxy measures) should be avoided where the children are able to self-report, and so the relevance and appropriateness of such input could be questioned [10, 11]. However, the focus of parent interviews in the present research was to assess the parent/caregiver’s perspective on their child’s ability to read and understand the items and the ease of incorporating the diary completion into their daily lives, rather than have parents act as proxy to provide responses to patient-reported concepts. Parents were able to observe their child during the at-home completion of the ePRO diary and consider their observations when responding to questions. Obtaining such parent/caregiver input during pediatric PRO development is typically recommended and considered best practice [12]. There is a debate in the literature regarding the ability of children aged 6–8 years to read and self-report health concepts, and their ability to do so typically depends on the concept and complexity of item wording [11, 12]. Moreover, children of that age range can be difficult to interview and can second-guess what the interviewer is asking. Thus, corroboration and input from parents regarding the ability of those children is considered valuable. However, it’s also important to stress that the parent input was considered secondary and supplementary to data directly reported by the children.

## Conclusions

The findings of the CE and CD interviews reported for this work provide evidence to support the content validity of a new ePRO instrument developed to assess the experience of chest congestion symptoms associated with the common cold experienced by children, adolescents, and adults.

## Supplementary Information

**Additional file 1.** Detailed Cognitive Debriefing Results.

## Data Availability

The qualitative data described in this article are not publicly available in further detail beyond that provided in the article and supplemental file but are available from the corresponding author on reasonable request.
