# Supplementary material for: Development and content validity testing of patient-reported outcome (PRO) items to assess chest congestion associated with the common cold for use in children and adolescents
Source: J Patient Rep Outcomes. 2022 May 28;6:56. doi: 10.1186/s41687-022-00465-8 (PMC9148330; doi:10.1186/s41687-022-00465-8)
Supplement: Supplementary file 1 — Additional file 1. Detailed Cognitive Debriefing Results. [file 41687_2022_465_MOESM1_ESM.docx]

# Supplementary File A: Detailed Cognitive Debriefing Results

This is a supplementary file for the manuscript titled: Development and content validity testing of patient-reported outcome (PRO) items to assess chest congestion associated with the common cold for use in children and adolescents

Authors: Rob Arbuckle, Chris Marshall, Laura Grant, Kate Burrows, Helmut Albrecht and Tim Shea

Submitted to The Patient.

## Instructions

All of the instructions were well understood by participants, and no issues were raised regarding the clarity of the instruction screens. Figure 20 below presents screenshots of all the instruction screens which guided participants between screens on the ePRO diary.


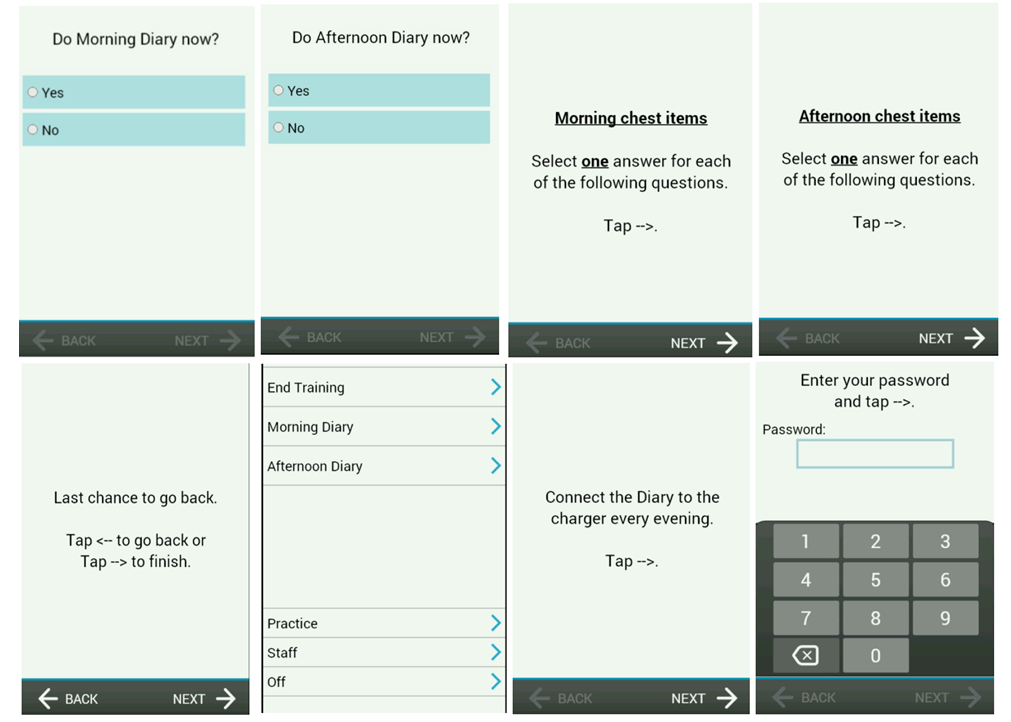


Figure 20. Instruction screens on ePRO diary

During the interim analysis of the round one interviews, there was concern that some participants were still thinking about their nasal symptoms when answering some items, rather than chest symptoms. To address this, a new instruction screen was added to the ePRO diary to remind participants to focus on their chest symptoms rather than nasal symptoms. The new instruction screen is presented in Figure 21.

Figure 21. New instruction screen tested in Round 2

All of the 23 participants interviewed in Round 2 were asked to provide feedback about the new instruction screen and all participants (23/23, 100%) understood this instruction, *“think about your chest area and not your nose, because those are very different things”* (0124-CC-F-11). In addition, six participants also mentioned that this instruction was a ‘helpful’ addition when answering the items.

## Concept: Difficulty breathing

Two items assessing difficulty breathing were debriefed with the participants; one on the ePRO (Figure 22) and one paper version (Figure 23). The wording of the two items was identical except that the paper version included ‘because of your cold’ at the end. Both items were well understood and equally relevant to participants.

### ePRO1: How hard was it to breathe air deep into your chest?


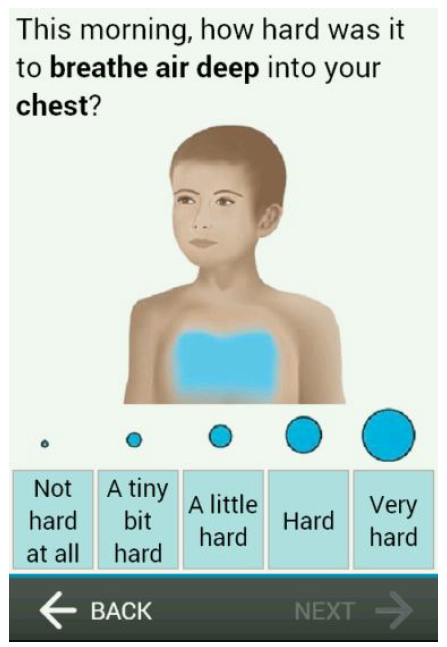


Figure 22. ePRO: Item 1 Understanding

Almost all of the participants (48/49, 98.0%) were asked about their level of understanding of item ePRO1. Thirty-eight of the 48 participants (38/48, 79.2%) demonstrated that they had understood this item, and were able to describe what the question was asking in their own words, *“Like breathing in and like, um, how hard was it to actually to like inhale”* (0210-CC-F-14).

Eight participants (8/48, 16.7%) misinterpreted the question or expressed that they didn’t quite understand what the item was asking, *“it’s like getting the stuff in your chest – it’s kind of like getting it all out”* (0205-CC-M-14). The majority of the participants who had difficulty understanding this item were in the younger age groups; seven participants were in the 6-8 age group and one participant was in the 12-17 age group. Of them, five participants had to ask the interviewer to explain the question, another participant interpreted this item as how physically ‘hard’ his chest felt (0112-CC-M-6), one participant thought the question was asking how difficult it was to breathe specifically through her nose (0117-RC-F-6) and the remaining participant thought the question referred to getting stuff out of his chest (0205-CC-M-14). In addition, it was unclear whether two of the participants had understood this item.

#### Relevance

Thirty-five out of the 48 participants (35/48, 72.9%) stated that this item related to a symptom that they had experienced during their cold, *“because I feel that I can’t take a deep breath yet all the way – without experiencing discomfort”* (0106-CC-F-53)*.* Thirteen participants (13/48, 27.1%) reported that they did not experience any difficulties breathing during their cold.

#### Reword

Of the 12 participants asked, three participants (3/12, 25.0%) reported that, given the opportunity, they would reword this question to make it easier to understand. One participant misinterpreted the item and related this item to ‘chest feeling stuffed’ (0119-CC-M-11), another participant found the item difficult to read (0206-CC-M-7) and another suggested removing the word ‘deep’ (0211-RC-M-17). Table 49 provides the quotes from these participants.

Table 49. Participant suggestions for item ePRO1 modifications

| **Suggestion** | **Quote** |
| --- | --- |
| Include the word ‘stuffed’ (n=1) | *“Um, this afternoon, did your chest feel stuffed at all?”* (0119-CC-M-11) |
| Use an easier word to read (n=1) | ***“Would you suggest a different word or?”*** *“Easier word to read.”* (0206-CC-M-7) |
| Remove the word ‘deep’ (n=1) | *“Maybe get rid of the word deep – because that could confuse people as to what you mean by deep versus just breathing.”* (0211-RC-M-17) |

### Paper1: How hard was it to breathe air deep into your chest because of your cold?


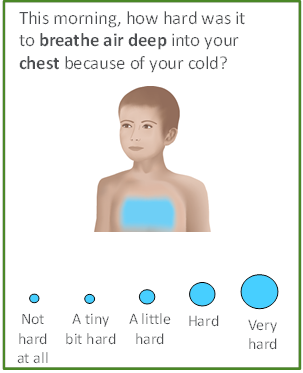


Figure 23. Paper: Item 1

#### Understanding

#### The paper and ePRO versions of ‘Item 1’ were almost identical, but the paper version included ‘because of your cold’ at the end. It was assumed that there would be a similar level of understanding for the ePRO and paper versions of this item, however, both items were debriefed separately. As this item had already been debriefed on the ePRO diary, and due to time constraints of the interview, the paper version was debriefed with slightly fewer participants than the ePRO item.

#### Thirty-two of thirty four participants asked (94.1%) showed a good level of understanding, “Like how hard was it to breathe, um, the air” (0116-CC-M-10). One participant in the 6-8 years age group did not understand this item, and when probed, he replied, “I forgot what it meant” (0120-CC-F-8). It was unclear whether one other participant had understood this item.

#### Relevance

Thirty-two of the 40 participants asked (32/40, 80.0%) reported that this item related to a symptom that they had experienced during their cold, *“um it was pretty hard… Some days it was harder than others though”* (0209-CC-F-16). Eight participants said that they did not experience difficulty breathing during their cold.

#### Reword

Eight participants were asked whether they would reword this item, and four of these participants (4/8, 50.0%) mentioned that they would modify the question to make it easier to understand. Two participants suggested removing the ‘because of your cold’ part of the item, one participant suggested making the item more concise (0104-CC-F-50), and another suggested making the item more precise by mentioning the lungs (0106-CC-F-53). Table 50 provides the quotes for these participants.

Table 50. Participant suggestions for item Paper1 modifications

| **Suggestion** | **Quote** |
| --- | --- |
| Remove additional words (n=2) | *“Because of – of your cold there, but, you know, I don’t – I don’t think it’s necessary.” (*0214-CC-F-11) |
| Make more concise (n=1) | *“Um, no, I mean a little wordy. I don’t know what I would take out, but it’s too long.”* (0104-CC-F-50) |
| Specify ‘lungs’ (n=1) | ***“Would you change it in any way?”*** *“Yeah. This morning how hard was it to take a – to take a deep breath into your chest because of your cold – how hard was it to take a deep breath into your lung.”*  (0106-CC-F-53) |

#### Parent feedback on ‘difficulty breathing’ items

All ten parents were debriefed on each of the items that assessed ‘difficulties breathing’. Only three parents stated that their children had reported difficulties with these items. Two of these parents had to explain the item to their child, *“I just said, OK, so think about how you have to breathe – (breath) all the way down to your bellybutton – I said that’s deep”* (0215-CC-M-6-P). The remaining parent stated their child needed help reading the item (0206-CC-M-7-P). In addition, one parent suggested removing the term ‘deep’ from this item as she reported that this had ‘confused’ her child (0128-CC-M-7-P).

## Concept: Chest tightness

Two items assessing chest tightness were debriefed with patients; one on the ePRO (Figure 24) and one paper version (Figure 25). The chest tightness items were well understood by most participants during CD, however, debriefing results from parents and children suggested some 6-11 year olds and some adolescents had difficulty understanding the term ‘tight’.

### ePRO2: How tight did your chest feel because of your cold?


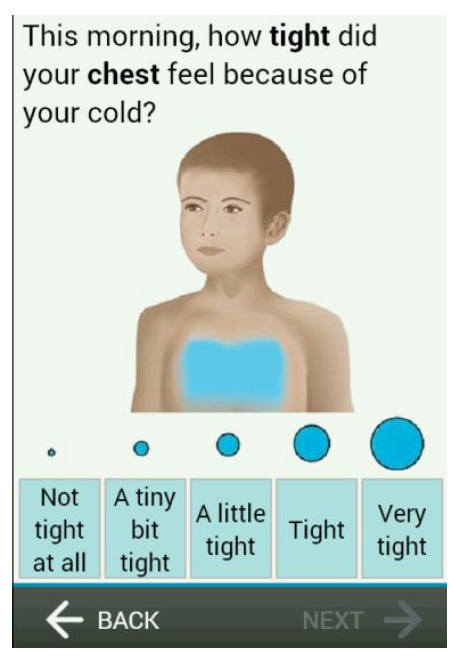


Figure 24. ePRO: Item 2

#### Understanding

All of the participants in the sample (49/49, 100.0%) were asked about their level of understanding of item ePRO2. Thirty-eight of these participants (38/49, 77.6%) showed a good level of understanding of chest tightness, *“like when your chest is like that – like you’re squeezing it”* (0126-CC-F-7).

Six participants (6/49, 12.2%) had difficulty understanding the item. All of the participants who struggled to understand this item were aged 17 years or younger: two participants were in the 6-8 age group, one participant was in the 9-11 age group, and three participants were in the 12-17 age group. Three of them admitted to not understanding the item, *“oh, this was one of the ones I didn’t understand the intention of – no clue”* (0211-RC-M-17). The remaining three participants misinterpreted the item; one participant referred to his stomach while explaining the item (0213-RC-M-6), one participant thought the item referred to how “smooshy” the chest felt (0217-RC-F-7), and one participant thought that the tightness referred to a noise (0109-RC-F-10). Due to the lack of detail provided, it was unclear whether five participants had understood the item.

#### Relevance

Forty out of the 45 participants asked (40/45, 88.9%) reported that this item related to a symptom that they had experienced during their cold, *“But I did feel it, like right when I got my cold”* (0126-CC-F-7). Five participants (5/40, 12.5%) reported that they did not experience any chest tightness during their cold.

#### Reword

Of the fourteen participants asked, only four participants (4/14, 28.6%) reported that they would make a change to the item. Two participants suggested replacing the word ‘tight’, one of which suggested using the term ‘closer together’. One participant felt that more explanation was needed for this item (0211-RC-M-17) and another participant provided alternative wording to describe the concept (0119-CC-M-11). Table 51 provides the quotes for these participants.

Table 51. Participant suggestions for item ePRO2 modifications

| **Suggestion** | **Quote** |
| --- | --- |
| Replace the word ‘tight’ (n=2) | *“I don’t know exactly what – the study is trying to get from the word tight – might be better to use another word.”* (0204-CC-F-45) |
| Improve the explanation (n=1) | *“I think it could’ve like explained that better.”* (0211-RC-M-17) |
| Alternative description (n=1) | *“Um, did you feel like you were in a compacted spot because of your cold?* (0119-CC-M-11) |

### Paper2: How tight did your chest feel?


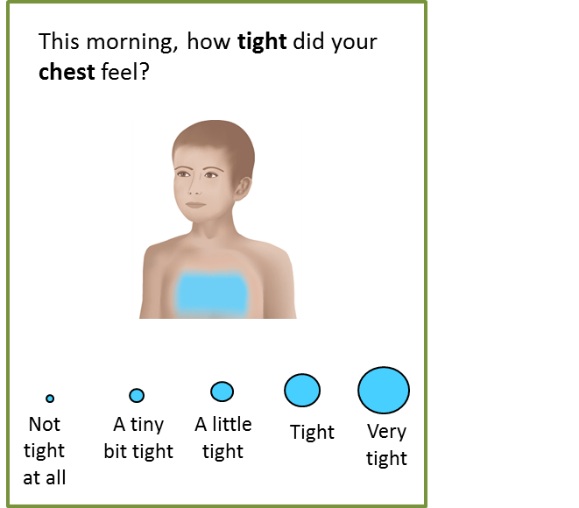


Figure 25. Paper: Item 2

#### Understanding

The paper and ePRO versions of the chest tightness item were almost identical, but the ePRO version included ‘because of your cold’ at the end. It was assumed that there would be a similar level of understanding for the ePRO and paper versions of this item however both items were debriefed separately. As this item had already been debriefed on the ePRO and due to the time constraints of the interview, the paper version was debriefed with fewer participants than the ePRO item.

Of the 31 participants that were asked, 28 participants (28/31, 90.3%) appeared to understand this item, *“compression maybe – a little tight – meaning it would be very hard – it would be quite hard to breathe”* (0123-CC-M-12). Only three participants (3/31, 9.7%; 2 adolescents and a 9 year old) had difficulty understanding chest tightness; two of these participants did not know the meaning of chest tightness and asked for an explanation (0211-RC-M-17 & 0121-CC-M-12) and one participant thought it referred to a *‘swelling’* (0202-RC-F-9).

#### Relevance

Thirty-two of the 49 patients (32/49, 65.3%) were questioned about the relevance of this item. Of these 32 participants, 18 participants (18/32, 56.3%) reported that this item was relevant to their cold, *“Oh, during my cold, yes I’ve had it”* (0106-CC-F-53). Thirteen participants (13/32, 40.6%) said chest tightness was not something that they had experienced, and it was unclear whether chest tightness was relevant for the remaining participant.

#### Reword

Only two of the 12 participants asked (2/12, 16.7%) reported that they would reword or change the item. One participant suggested changing the term chest tightness to the term ‘closed in’ (0116-CC-M-10), while the other participant stated that ‘compression’ would be a more accurate description of chest tightness (0123-CC-M-12).

#### Parent feedback on ‘chest tightness’ items

When discussing the chest tightness items, five parents stated that their child either did not recognize or understand the word ‘tight’, *‘um, tight, I think, is a little rough word for him. I don’t think he exactly understands what type – tight is, but maybe just a little bit’* (0120-CC-F-8-P). One parent suggested it may be more helpful to talk about the feeling of chest tightness more broadly, and ask *‘when you breathe in, do you feel anything?’* (0213-RC-M-6-P) rather than using a complex description. Another parent suggested it would be more helpful to include an explanation of tightness, to help the child understand the term ‘tight’, *‘Maybe put it somewhat like – like a towel wrung together – to give an example of how something feels tight – some kind of visual like that might be better’* (0128-CC-M-7-P).

## Concept: Chest pain

Prior to the interviews, chest pain was not understood to be a relevant symptom associated with chest congestion and therefore was not included as a topic of interest in the original interview guide. However, during round one interviews almost half of the sample spontaneously discussed chest pain as a symptom of their cold and so this symptom was added to the interview guide as a topic for discussion in the second round of interviews. Two items assessing chest pain were debriefed with participants; one on the ePRO (Figure 26) and one paper version (Figure 27). Both items assessed chest pain in relation to another chest symptom (i.e. chest hurt due to coughing and chest hurt due to being stuffed up). Chest pain due to coughing was well understood and relevant to the majority of participants, whereas chest pain due to being stuffed up was poorly understood across the age groups mainly due to difficulty understanding ‘stuffed up’.

### ePRO3: How much has your chest hurt when you’ve coughed?


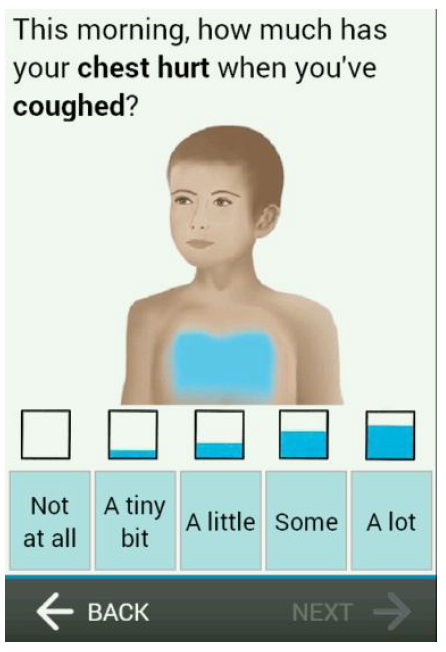


Figure 26. ePRO: Item 3

#### Understanding

All of the participants (49/49, 100.0%) were probed on their understanding of this item. Forty-two of these participants (42/49, 85.7%) demonstrated understanding of the item and experienced no difficulties answering or selecting a response, *‘it means like whenever you cough, your chest feels like a hurting, like an aching’* (0113-CC-M-9).

Six of the 49 participants (6/49, 12.2%) did not understand the item. The participants who did not understand the item were mostly from the younger age groups; three participants were in the 6-8 year old age group, two participants were in the 9-11 years age group and one participant was in the adult age category. Four participants stated that they were not able to comprehend the item, *‘um, I’m not sure’* (0122-RC-F-11), one participant referred to his ‘stomach’ when explaining the question (0213-RC-M-6), and the remaining participant thought that the question related to breathing difficulties, *‘it means, did you have a lot of problems breathing’* (0118-CC-M-74).

#### Relevance

Thirty-nine of the 44 participants asked (39/44, 88.6%) stated that their chest hurt when they coughed during their cold, *“like if there’s – anything in your chest and you cough – it might hurt”* (0132-CC-M-16). Three participants did not experience this symptom during their cold (3/39, 7.7%), and it was unclear whether or not it was relevant for two participants.

#### Reword

Of the sixteen participants asked, only one participant (1/16, 6.3%), aged 50 years old, stated that she would modify this item to make it clearer, *“make it make more sense – is how much does you – body hurt from coughing”* (0203-CC-F-50).

### Paper3: How much has your chest hurt due to being stuffed up?


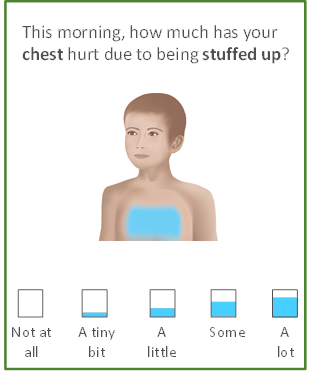


Figure 27. Paper: Item 3

#### Understanding

Forty-one of the 49 patients (41/49, 83.7%) discussed their level of understanding of this item. Of these 41 participants, 30 participants (30/41, 73.2%) clearly understood the item and were subsequently able to explain the item in their own words, *‘um I think that means, like, um, if your – stuffed up on the inside with like, mucus or something in there, um, then you’ll start feeling pains in there that will hurt you’* (0119-CC-M-11).

Eleven of the 41 participants (11/41, 26.8%) had difficulties understanding this item. Eight of these participants stated that they had not understood and asked the interviewer for further clarification, two of the participants were not sure whether the phrase ‘stuffed up’ referred to their chest or nose (0122-RC-F-11; 0204-CC-F-45), and one participant thought it meant *‘stuffed up with food’* (0120-CC-F-8).

#### Relevance

Of the 35 participants asked, only 17 participants (17/35, 48.6%) reported that this question related to a symptom that they had experienced during their cold, *“oh during my cold? I’m going to say a little”* (0106-CC-F-53). Eleven participants did not experience this symptom during their cold (11/35, 31.4%), and it was unclear whether seven participants had experienced any chest pain due to being stuffed up. This was either because the participant focused their response on the understanding of the item, or their response did not provide enough detail to establish the relevance of the symptom.

#### Reword

Sixteen participants were asked whether they would change the item. Half of the participants asked (8/16, 50.0%) confirmed that they would change something about the wording of the item. The majority of participants suggested clarifying the item by either removing or changing the wording for ‘stuffed up’. Table 52 provides some of the quotes for these participants.

Table 52. Participant suggestions for item Paper3 modifications

| **Suggestion** | **Quote** |
| --- | --- |
| Remove phrase ‘stuffed up’ (n=2) | *“The word stuffed just kind of like that could be anything.”* ***“Is there another word that you would use instead?”*** *“Um, so like – like full of something but I don’t know.”* (0210-CC-F-14) |
| Paraphrase item (n=2) | *“I would just say, this afternoon, how much has your chest hurt due to your cold?”* (0105-CC-M-58) |
| Change the tense of the wording (n=1) | *“Um, I think that you should change had as in had or if the – uh, never mind – because it’s talking about right now.”* (0102-CC-F-11) |
| Alternative word for ‘stuffed up’ (n=1) | *“I think they should’ve used, like, the word, you know, phlegm or, um, goo like they said it before.”* (0104-CC-F-50) |
| Alternative wording for ‘hurt’ (n=1) | *“Or perhaps the question should rather be like how – like I think- Did you experience any chest pain?”* (0132-CC-M-16) |
| Clarify where in the body is ‘stuffed up’ (n=1) | *“You might want to clarify that – because – especially younger kids wouldn’t get that connection – the other body parts being stuffed up versus the chest being stuffed up.”* (0211-RC-M-17) |

#### Parent feedback on ‘chest pain’ items

Parents were debriefed on the items representing the concept of ‘chest pain’, and subsequently discussed whether they thought that their child had understood each item. Four parents reported that their children had not understood at least one of the items assessing chest pain. One parent stated that item Paper3 was a little too wordy (0126-CC-F-7-P), another parent stated that their child was confused by the ‘hurt when coughed’ phrase of item ePRO3 (0215-CC-M-6-P), and the remaining parent stated that his child had not been able to comprehend the ‘when stuffed up’ terminology of item Paper3, *“so I mean the stuffed up, he might – he got a little confused on that anyway”* (0131-CC-M-6-P). The remaining parent did not specify why her child had difficulty understanding the item (0213-RC-M-6-P). One parent suggested removing the term ‘stuffed up’ (0131-CC-M-6-P), and another parent suggested rephrasing this item to, *“did you feel any pain – did anything hurt in your chest?”* (0213-RC-M-6-P)

## Concept: Heavy chest

Although only eight participants (8/49, 16.3%) reported this symptom spontaneously during CE, 35 participants (35/49, 71.4%) reported that this was a relevant symptom of their cold during CD. Two items assessing chest heaviness were debriefed with participants; one on the ePRO (Figure 28) and one paper version (Figure 29). One item asked directly about the level of heaviness experienced in the chest and the other asked about the amount of time that the chest felt heavy. Both items performed similarly in the CD interviews.

### ePRO4: How heavy did your chest feel?


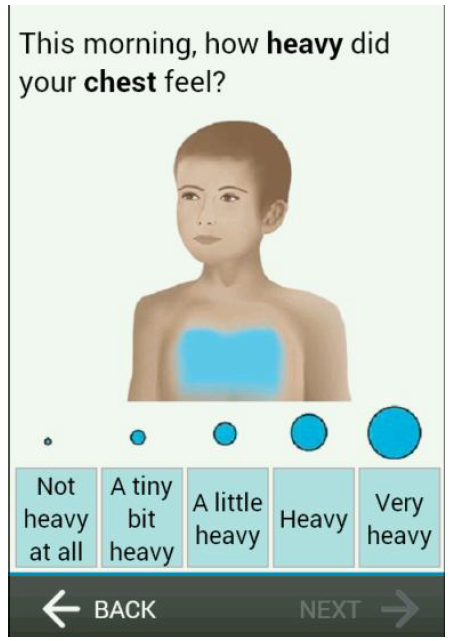


Figure 28. ePRO: Item 4

#### Understanding

Forty-eight of the 49 participants (48/49, 97.6%) discussed their level of understanding of this item. Thirty-two of the 48 participants (32/48, 66.7%) appeared to understand this item, *‘heavy means it’s kind of like, I guess pulling you down’* (0205-CC-M-14).

Eight of the 48 participants did not understand this item (8/48, 16.7%). Five of these participants simply did not understand the item, *“I didn’t get the intention or just like I said, I didn’t know your chest could change weight”* (0211-RC-M-17). One participant referred to the heaviness as residing in their ‘belly’ (0213-RC-M-6), one participant thought that it meant that their walking was affected (0125-CC-F-8), and the final participant associated this item to coughing and a popping movement (0110-CC-F-15). A further eight participants did not make it clear enough during the interview to determine whether they had understood the item. This was generally a problem for the younger participants who were unsure whether they had experienced the symptom and it was therefore difficult to assess whether or not the participants had understood the item.

#### Relevance

Thirty-five of the 46 participants asked (35/46, 76.1%) reported that having a ‘heavy’ chest was something that they had experienced during their cold. When responding to this item, one participant responded *‘not heavy at all. But when I had my cold, just a little bit’* (0109-RC-F-10). Nine participants (9/35, 25.7%) reported that they did not experience any chest heaviness during their cold. For two participants, it was not clear whether or not they had experienced a heavy chest.

#### Reword

Of the thirteen participants asked, four participants (4/13, 30.8%) mentioned that they would reword this item. The majority of suggestions included modifying the term ‘heavy’ or providing an alternative description of chest heaviness. Table 53 provides some of the quotes for these participants.

Table 53. Participant suggestions for item ePRO4 modifications

| **Suggestion** | **Quote** |
| --- | --- |
| Specify heaviness (n=1) | *“I would rephrase the question – heavy, meaning mucus or heavy meaning – this morning, how heavy did my chest feel. Full of mucus or -?”* (0106-CC-F-53) |
| Combine heavy with tight (n=1) | *“I would combine a couple of them. Like the one that said tight and the one that said heavy – that’s pretty much the same thing.”* (0108-CC-M-51) |
| Remove term ‘heavy’ (n=1) | *“That should be a little more self-explanatory – because a heavy chest, uh, means you could be having a heart attack – I don’t know what word I could use in place, but I don’t think heavy’s the – word to use.”* (0118-CC-M-74) |
| ‘Congested’ rather than ‘heavy’ (n=1) | *“I mean I would just say, instead of heavy, maybe congested.”* (0204-CC-F-45) |

### Paper4: How much of the time has your chest felt heavy?


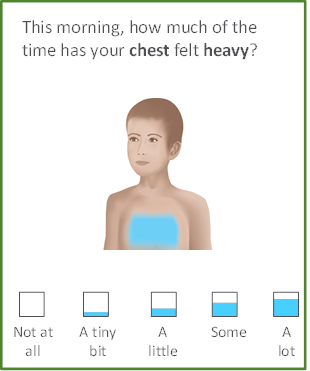


Figure 29. Paper: Item 4

#### Understanding

Thirty-five out of the 49 (35/49, 71.4%) participants were debriefed on this item. Of these, 21 participants demonstrated good level of understanding of this item (21/35, 60.0%), *‘well this is a little bit different than the other ones, because it’s asking for an amount of time that it’s felt heavy’* (0214-CC-M-58). Ten participants did not understand this item; seven of these stated that they were not sure what the item was asking, two of these referred to ‘heavy’ being as full of food (0211-RC-M-17 & 0213-RC-M-6), and one participant described the heaviness as affecting the ability to walk (0125-CC-F-8). Five of the seven participants who reported that they were not sure what the item was asking were in the two youngest age groups (6-8; 9-11 years old), suggesting that this concept may have been slightly more difficult for the younger participants to understand. The remaining four participants did not make it clear whether or not they had understood this item. Two of these participants who did not make it clear whether or not they had understood were seven years old, one participant was nine years old and the remaining participant was 45 years old.

#### Relevance

Thirty-one of the 49 participants (31/49, 63.3%) were asked about item relevance. Of these, 20 participants (20/31, 64.5%) reported that they had experienced a heavy chest during their cold whereas eight participants (8/31, 25.8%) stated that this was not a relevant symptom for them. It was unclear whether or not this was relevant for three participants due to an inadequate detail provided by participants.

#### Reword

Eleven participants (11/49, 22.4%) were either asked or spontaneously commented on whether this item would benefit from being modified. Five of the eleven participants (5/11, 45.5%) recommended changing this item in order to make it easier to understand. Of these participants, the majority suggested simplifying the item or providing an alternative description to improve participant understanding. Table 54 provides some of the quotes from these participants.

Table 54. Participant suggestions for item Paper4 modifications

| **Suggestion** | **Quote** |
| --- | --- |
| Specify time (n=2) | *“Maybe you could say like in minutes or hours, because there’s no definition of, you know, how long.”*(0204-CC-F-45) |
| Change recall (n=1) | *“Overnight, you know, you’re sleeping. I think it should’ve said something like – like, you know, how hard was it to sleep last night – or did it keep you up or something.”* (0104-CC-F-50) |
| Change to ‘how long’ (n=1) | *“That’s a weird way to say it. A better way’d be like how long has your chest felt heavy? Like how much of the time? That sounds like a translation.”* (0132-CC-M-16) |
| Simplify item (n=1) | ***“Is there anything you’d change about this question?”*** *“Uh, it’s kind of complicating like what it actually is asking.”* (0211-RC-M-17) |

#### Parent feedback on ‘chest heaviness’ items

Seven of the ten parents (7/10, 70.0%) stated that their child struggled to understand the concept of a ‘heavy’ chest, *‘so I think that’s an – that’s the other one that’s a little difficult for him to understand’* (0120-CC-F-8-P). Several parents made a suggestion regarding how the items could be amended in order to improve their children’s understanding. One parent suggested changing the item to, *‘could you feel the mucus in there when you coughed?’* (0213-RC-M-6-P), another parent suggested including a description of chest heaviness and provide an example of how ‘heaviness’ would feel, *‘does it feel like there’s something heavy that’s laying on your chest – something that gives them a reference of what feels like it’s on your chest?’* (0215-CC-M-6-P) Another parent suggested rephrasing the item and including the example of being *‘weighed down’* (0128-CC-M-7-P). Of note, not all parents who reported that their child had a problem understanding the item suggested any changes.

## Concept: Chest feels full

Seven items assessing the concept of ‘chest feels full’ were debriefed with participants. Following round one, the wording of item ePRO5 was amended and the new wording was tested in round two. The item ePRO5 which was tested in round one is presented in Figure 30 and the amended item tested in round two is presented in Figure 31. Screenshots of the five other items assessing the chest feels full concept are presented in: Figure 32, Figure 33, Figure 34, Figure 35, Figure 36 and Figure 37. The newly added item ePRO5d was poorly understood by almost a third of the participants. The item ePRO5 performed equally well with the term ‘goo’ rather than ‘the goo that comes out of your nose’. Both items measuring the symptom ‘clogged up’ performed similarly in terms of level of understanding and relevance. Both items measuring the symptom ‘stuffed up’ also performed similarly in terms of the level of understanding and relevance.

### ePRO5 (Round 1): How much did your chest feel full of mucus (the goo that comes out of your nose)?


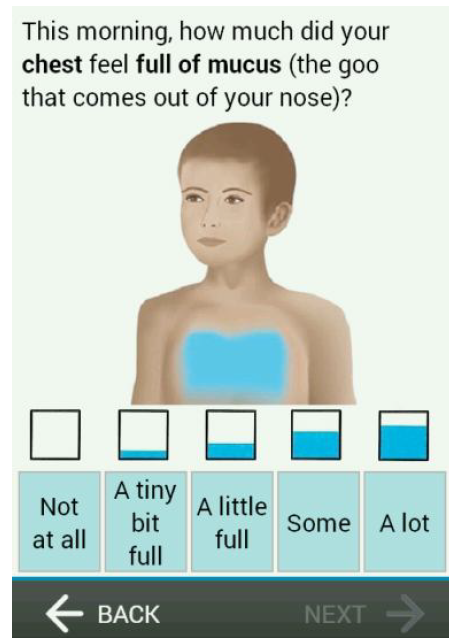


Figure 30. ePRO 5 (Round 1): Item 5

#### Understanding

This item was only debriefed during the first round of interviews as it was modified following the interim analysis for the second round of interviews. Therefore, this item was debriefed with the 26 participants in round one. Twenty-one of these 26 participants (21/26, 80.0%) understood this item, and were able to respond accordingly, *‘I understand by mucus as in like that gooey stuff that you cough up when like you’re – when you have really bad cos – congestion in your lungs and throat’* (0102-CC-F-11). Five participants (5/26, 19.2%) struggled to understand this item, of whom, three participants did not understand the concept of the chest being full of mucus, and the remaining two participants associated this item with their nose being full of mucus, *‘like when your nose is really runny’* (0206-CC-M-7). Of the participants who did not understand this item, three of the participants were in 6-8 years age group, and two of the participants were in the 12-17 years age group.

#### Relevance

Of the 26 participants asked, 20 of these participants (20/26, 76.9%) specifically said that this question related to a symptom that they had experienced during their cold, *‘because there’s not a lot, but there’s some of it’* (0201-CC-F-11). The remaining six participants (6/26, 23.1%) reported that their chest did not feel ‘full of mucus’ or ‘goo’ during their cold.

#### Reword

Of the 14 participants who were asked, six participants (6/14, 42.8%) reported that they would reword this item in order to make it clearer or easier to understand. Of these participants, the majority suggested modifying or removing the term ‘mucus’, and/or providing a clearer description of ‘mucus’. Table 55 displays the different suggestions proposed by the participants.

Table 55. Participant suggestions for item ePRO5 (Round 1) modifications

| **Suggestion** | **Quote** |
| --- | --- |
| Specify where the mucus comes out (n=1) | *“I’d probably say, uh, how much did your chest feel full of mucus and phlegm coming out of your nose and mouth.”* (0103-CC-F-70) |
| Include a clearer description of mucus (n=1) | *“But maybe in parentheses following mucus – have a little descriptor or something – might be helpful”* (0107-RC-M-8) |
| Replace the word ‘mucus’ with a more child-friendly term (n=1) | *“If you’re doing this for kids – I don’t think they would understand what mucus was – I think he’d know what – you know, snot coming out of your nose, or you know, spit coming out of your throat.”* (0203-CC-F-50) |
| Specify the feeling of being full of mucus (n=1) | *“I found it hard to think what the difference would be between having your chest full of something and full of mucus – maybe if like when you.”* (0208-RC-M-13) |
| Rephrase the item (n=1) | ***“If you had to put this question into your own words, how would you do that?”*** *“Um – like how much did your, um – for me, it feels like in my nose still and like – um, in my throat – how much did that feel like you had like – like still, um, in you.”* (0210-CC-F-14) |
| Combine ‘full of mucus’ and ‘stuffed up’ (n=1) | *“I think you should word it where it has this morning, how stuffed up or full of mucus did your chest feel – And just combine them.”* (0211-RC-M-17) |

### ePRO5 (Round 2): How much did your chest feel full of mucus (goo)?

Figure 31. ePRO 5 (Round 2): Item 5

#### Understanding

Following round one, the description in the parentheses was removed and changed from ‘the goo that comes out of your nose’ to just ‘goo’. This was based on several children during the first round of interviews focusing on nasal symptoms as opposed to chest symptoms, and so any reference to nasal symptoms was removed for consistency and clarity. This modified item was then tested with the 23 participants in the second round of interviews. Twenty out of the 23 participants asked (20/23, 87.0%) appeared to understand this item, confirming that the additional detail included in the parentheses in round one was of no additional benefit and may have confused. For example*,* when asked what one participant understood by this question, she responded, *‘It means like spit and boogers’* (0120-CC-F-8).

Three of the participants (3/23, 13.0%) reported difficulty understanding this item and required help to respond to the item: *‘This is the one my mum had to help me with’* (0125-CC-F-8). All three participants who had problems understanding this item were in the 6-8 year old age group.

#### Relevance

Thirteen of the 20 participants asked (13/20, 65.0%) reported that this was a symptom they experienced during their cold. One participant mentioned that they felt *‘a little full – like the first day’* (0121-CC-M-12). Five participants (5/20, 25.0%) reported that this item was not relevant as their chest was not full of mucus during their cold, *‘Because there was no mucus in me’* (0212-RC-M-7). It was not clear whether this symptom was relevant for two of the participants.

#### Reword

Four of the six participants asked (4/6, 66.7%) stated that they would reword this item. All of these participants suggested removing and/or replacing certain words with alternative, simpler terms. Table 56 provides some of the quotes from these participants.

Table 56. Participant suggestions for item ePRO5 (Round 2) modifications

| **Suggestion** | **Quote** |
| --- | --- |
| Remove the word ‘goo’ (n=2) | *“Uh, I don’t use the word goo. Mucus or like snot – I’d just use snot.”* (0129-CC-M-11) |
| Replace ‘full’ with ‘stuffed’ (n=1) | *“Um this afternoon, did your chest feel stuffed at all?”* (0119-CC-M-11) |
| Replace ‘mucus’ with ‘goo’ (n=1) | *“Replace mucus with goo.”* (0123-CC-M-12) |

### ePRO6: How stuffed up did your chest feel?


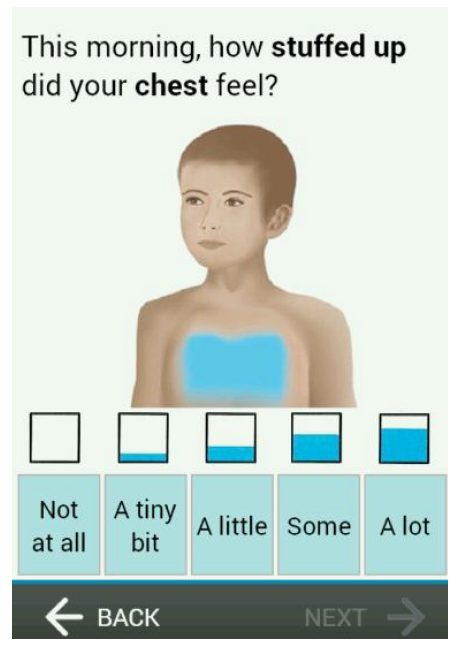


Figure 32. ePRO: Item 6

#### Understanding

Almost all of the participants across both rounds of interviews (47/49, 96.0%) reported on their level of understanding of this item. Thirty-seven of the 47 participants asked (37/47, 78.7%) demonstrated that they had definitely understood this item, ‘*I understand this question that it’s asking me how much phlegm or mucus was – that I could this that there was – that there was in my chest.’* (0124-CC-F-11)

Eight participants (8/47, 17.0%) did not understand this item. Two participants reported that this item referred to their stomach and being ‘hungry’, one participant thought that this item referred to nasal congestion (0112-CC-M-6), one participant though it meant ‘stuffed up’ with air (0131-CC-M-6), one participant thought it meant ‘stuffed up’ with dust (0111-CC-M-12), and the remaining three admitted to simply not understanding the item, *“Um, this I didn’t really understand – I don’t even know”* (0210-CC-F-14). Four of these participants were in the 6-8 years age group, one participant was in the 9-11 years age group, and the remaining three participants were in the 12-17 years age group. The remaining two participants did not make it clear whether or not they had understood the item.

#### Relevance

Of the 45 participants asked, 35 participants (35/45, 77.8%) reported that feeling ‘stuffed up’ was a symptom that they had experienced during their cold, *‘Because I am a little stuffed up from my chest’* (0213-RC-M-6). Seven participants (7/35, 20.0%) stated that they hadn’t felt ‘stuffed up’ during their cold, and it was unclear whether this was a relevant symptom for three participants.

#### Reword

Of the 18 participants who were asked, nine of these participants (9/18, 50.0%) specified that they would reword the question. Common suggestions from these participants were to replace words, add phrases and/or combine with other items. Table 57 displays the suggestions made by different participants.

Table 57.Participant suggestions for item ePRO6 modifications

| **Suggestion** | **Quote** |
| --- | --- |
| Replace ‘stuffed’ with ‘congested’ (n=2) | *“I don’t understand the – chest being stuffed up – well from the question, I understand more of how congested you feel.”* (0207-CC-M-26) |
| Shorten the item & do not specify chest (n=1) | *“I would rephrase the question. How stuffed up did you feel.”* (0106-CC-F-53) |
| Combine with other questions (n=1) | *“So you know, they could reduce it, because you’re – you’re asking the same thing.”* (0108-CC-M-51) |
| Replace ‘stuffed’ with ‘hard to clear’ (n=1) | *“Um, maybe I would use hard to clear.”* (0109-RC-F-10) |
| Replace ‘stuffed’ with ‘hard to breath’ (n=1) | *“I would say like – my nose is kind of stuffed up, like I don’t – it’s kind of different with your chest.* ***“Is there anything else that you would use?”*** *“Just like hard to breathe, I guess.”* (0114-RC-F-14) |
| Replace ‘stuffed’ with ‘full’ (n=1) | ***“Is there another word that you would say instead of that?”*** *“Full.”* (0202-RC-F-9) |
| Replace ‘stuffed’ with ‘closed up’ (n=1) | *“See again, when you say stuffed up. I’m thinking of my nose – How closed up does your chest feel – rather than stuffed up, I would use.”*(0203-CC-F-50) |
| Add ‘full of mucus’ (n=1) | *“I think you should word it where it has this morning, how stuffed up or full of mucus did your chest feel.”* (0211-RC-M-17) |

### Paper5a: How much did you feel stuffed up in your chest?


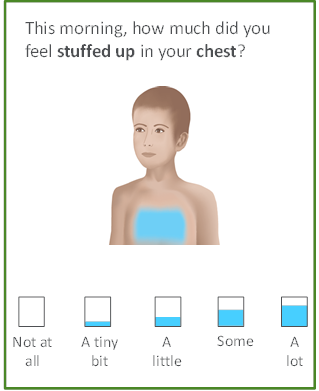


Figure 33. Paper: Item 5a

#### Understanding

Forty-one of the 49 participants (41/49, 83.7%) reported their level of understanding of this item. Of these 41 participants asked, 26 participants (26/41, 63.4%) showed a good level of understanding of the item, *‘Uh, you have a lot of stuff in your chest – it could be mucus or something like that’* (0208-RC-M-13).

Eight participants (8/41, 19.5%) had difficulty understanding this item. Three of these participants discussed their stomach or being hungry in relation to the item, *“Like how full in my stomach”* (0217-RC-F-7). One participant referred to nasal congestion when interpreting the item (0101-CC-F-8), one participant thought that the phrase ‘stuffed up’ meant stuffed up with ‘dust’ (0111-CC-M-12) and the remaining three participants reported that they were not sure what the item meant. The participants who had difficulty understanding the item were from a variety of age groups: five were in the 6-8 years age group, one participant was in the 9-11 years age group, one participant was in the 12-17 years age group and one participant was in the adult category. In addition, seven participants had not made it clear whether or not they had understood the item.

#### Relevance

Over half of the participants asked reported that feeling ‘stuffed up in the chest’ was a relevant symptom. Sixteen of the 29 participants asked (16/29, 55.2%) specifically said that they had felt ‘stuffed up’ in their chest at some point during their cold, *‘Because I was stuffed up a little bit’* (0213-RC-M-6). Eight participants said that they had not experienced this symptom (8/29, 27.6%) and it was unclear whether or not five participants had experienced the symptom.

#### Reword

Four of the 11 participants asked (4/11, 36.4%) stated that they would in some way change the question to improve clarity. The majority of participants chose to change the wording for ‘stuffed up’ and suggested an alternative term. Table 58 provides some of the quotes from these participants.

Table 58. Participant suggestions for item Paper5a modifications

| **Suggestion** | **Quote** |
| --- | --- |
| Reword & simplify item (n=2) | *“I don’t like the wording on this at all – the wording is awkward – I would say this afternoon, how stuffed up did your chest feel?”* (0105-CC-M-58) |
| Replace ‘stuffed up’ with ‘congested’ (n=1) | *“That’s a no-no. OK. This morning, how much did you feel congested in your chest – your chest doesn’t feel stuffed.”* (0106-CC-F-53) |
| Remove ‘stuffed up’ (n=1) | ***“What would you change about this – to make it easier to understand?”*** *“Probably like a different word – like different words than stuffed up.”* (0120-CC-F-14) |

### Paper/ePRO5b: How much did you feel clogged up in your chest?

Figure 34. Paper/ePRO: Item 5b

Following the first round of interviews and interim analysis, item 5b performed well when debriefed on paper and as a result, this item was then programmed onto the ePRO device for testing in the second round of interviews. As the wording of the item remained the same, the results have been collated from across round one and round two for the purposes of analyses.

#### Understanding

The majority of the participants were asked whether or not they had understood this item (44/49, 89.8%). Thirty-six of these participants (36/44, 81.8%) showed no difficulties comprehending or answering the item, *‘um, how much mucus was stuck in your chest?’* (0202-RC-F-9)

Six participants (6/44, 13.6%) experienced difficulties understanding this item and asked the interviewer for help. All of these participants were in the 6-8 year old age group. One participant thought that this question related to the stomach being clogged up (0213-RC-M-6), and the remaining five participants reported to not know what the word ‘clogged’ meant. It was not clear whether or not the remaining two participants had understood the item.

#### Relevance

Of the 36 participants asked, 24 participants (24/36, 66.7%) explained to the interviewer that, at some point during their cold, they had felt clogged up in their chest, *‘I was pretty stuffed and clogged’* (0103-CC-F-70). Six participants (6/36, 16.7%) reported that they had not experienced feeling ‘clogged up’ during their cold, and for the remaining six participants, there was not enough evidence from the interviews to establish whether it was a relevant symptom.

#### Reword

Despite a relatively high level of understanding amongst participants, nine out of the 12 participants asked (9/12, 75.0%) stated that they would reword the question. The most frequently reported suggestion was to reword the term ‘clogged up’. This provides evidence that ‘clogged up’ is not as good as other wording tested. Table 59 provides an overview of the suggestions made by participants.

Table 59. Participant suggestions for item Paper/ePRO 5b modifications

| **Suggestion** | **Quote** |
| --- | --- |
| Replace ‘clogged up’ with ‘stuffed up’ (n=3) | *“Like I would say, um clogged up – well I don’t really know another way. But I might think of one – stuffed up.”* (0126-CC-F-7) |
| Replace ‘clogged up’ with ‘congested’ (n=2) | *“Clogged up has to go – the proper word, congested.”* (0106-CC-F-53) |
| State the cause of being ‘clogged up’ (n=1) | *“So, you know, it should be – this morning how much did your chest feel clogged from being overnight – or from the build up from overnight.”* (0104-CC-F-50) |
| Enquire about lung functioning (n=1) | *“Did your chest feel like your lungs were not working?”* (0119-CC-M-11) |
| Replace ‘clogged up’ with ‘full of mucus’ (n=1) | ***“Are there any other words you would use instead of that?”*** *“Like is there like mucus, I guess?”* (0121-CC-M-12) |
| Replace ‘clogged up’ with ‘blocked up’ (n=1) | ***“Is there any other word or term you would use instead of clogged up?”*** *“Um, maybe blocking up.”* (0208-RC-M-13) |

### Paper/ePRO5c: How clogged up did your chest feel?

Figure 35. Paper/ePRO: Item 5c

Similar to item 5b (‘how much did you feel clogged up in your chest’), item 5c was found to be well understood and relevant when debriefed on paper during the first round of interviews, and subsequently was programmed onto the ePRO diary for testing in electronic format during the second round of interviews. Again, the data from both rounds have been collated for the purposes of analyses.

#### Understanding

Twenty-eight of the 34 participants asked (28/34, 82.4%) appeared to understand this item and were subsequently able to provide a description of this symptom, *‘clogged up means that you – that you’re like coughing up like weird greenish yellow stuff from your throat’* (0102-CC-F-11).

Six participants (6/34, 17.6%) had difficulty understanding this item. Two of these participants, once again, referred to their stomach as being clogged (0122-RC-F-11 & 0213-RC-M-6) and the remaining four participants reported that they were not sure what the item was asking. Of the participants who had difficulty understanding this item, five participants were in the 6-8 years age group and the remaining participant was in the 9-11 years age group.

Of note, the same participants had difficulty understanding all the items in the ‘chest feels full’ concept and the same rationale was used to describe the aspect of the item which the participant did not understand (e.g. referring to the stomach).

#### Relevance

The relevance of the term ‘clogged up’ had already been assessed in ‘Item 5b’ and therefore fewer participants were debriefed on the relevance of this item. Sixteen of the 27 participants asked (16/27, 59.3%) reported that they had felt clogged up in their chest during their cold, *‘how clogged up did your chest feel? Um, I have felt this before in my cold’* (0124-CC-F-11). Nine participants (9/27, 33.3%) reported that they had not felt clogged up during their cold, and it was unclear whether this item was relevant for two participants.

#### Reword

Three of the five participants asked (3/5, 60.0%) mentioned that, given the opportunity, they would reword the item. Of these participants, it was commonly suggested to modify the wording for ‘clogged up’ to improve understanding. Table 60 provides a description of how the participants would reword this item.

Table 60. Participant suggestions for item Paper/ePRO 5c modifications

| **Suggestion** | **Quote** |
| --- | --- |
| Replace ‘clogged up’ with ‘stuffed up’ (n=1) | *“This morning how much did you feel stuffed up in your chest? And eliminate the other two.”* (0103-CC-F-70) |
| Replace ‘clogged up’ with ‘congested’ (n=1) | *“The proper word is congested.”*(0106-CC-F-53) |
| Further clarify item (n=1) | *“I guess as long as you have something under it, like a little bit, a lot to clarify.”* (0204-CC-F-45) |

### Paper6c: How clear did your chest feel?


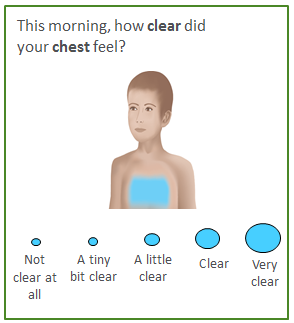


Figure 36. Paper: Item 6c

#### Understanding

Twenty-nine of the 39 participants asked (29/39, 74.4%) appeared to understand this item and experienced no difficulties in answering the item, *‘like not congested or blocked or stuffed up. Just kind of like empty and normal’* (0209-CC-F-16). Seven participants did not understand this item (7/39, 17.9%) and required further clarification before being able to answer. Five participants expressed that this item was ‘vague’, and that they were unable to make sense of the question, *‘I don’t even know what that one means, yeah’* (0120-CC-F-8). One participant thought that it meant how ‘clear’ their stomach was (0122-RC-F-11), and another participant answered the question in relation to his throat (0206-CC-M-7). Four of the participants who had difficulty understanding this item were in the 6-8 years age group, one participant was in the 9-11 years age group and the remaining participant was in the adult age category.

#### Relevance

Of the 31 participants asked, 23 participants (23/31, 74.2%) stated that ‘clearing their chest’ was a relevant symptom of their cold, *‘Uh during the day, I felt more clear than at night’* (0103-CC-F-70). Three participants (3/31, 9.7%) reported to not experience this symptom. It was unclear whether or not this was a relevant symptom for five participants.

#### Reword

Four participants were asked whether or not they would change this item to make it easier to understand. Two of these four (2/4, 50.0%) suggested making small modifications. One patient suggested combining this item with a previous item, *‘combine something – but not make it too wordy either – so it’s kind of tough’* (0104-CC-F-50). The other participant suggested changing the wording so that the response scale would not be reversed in comparison to the other items (0118-CC-M-74).

### ePRO5d: How full of stuff did your chest feel?

Figure 37. ePRO: Item 5d

During the first round of interviews, some of the participants referred to the concept of ‘stuffed up’ as feeling ‘full of stuff’. Therefore, following the interim analysis, an item assessing ‘full of stuff’ was programmed onto the ePRO diary in order to test this alternative wording.

#### Understanding

As this was a new item, it was only debriefed with the 23 participants in the second round of interviews. All 23 participants were asked about their level of understanding of this item. Of these, 15 participants (15/23, 65.2%) appeared to understand this item, ‘*I understand this question by asking how much phlegm or mucus there was in my chest?’* (0127-CC-F-11). Six participants (6/23, 26.1%) struggled to understand this item. Two of these participants thought that this item referred to the *‘stomach’* and *‘food’* (0122-RC-F-11 & 0217-RC-F-7), one participant referred to ‘full of stuff’ as *‘dirt or sand’* (0120-CC-F-8) and the remaining three participants stated that they were not sure what this meant. Most of the participants who had difficulty understanding this item were in the younger age groups; five participants were in the 6-8 year old age group and one participant was in the 9-11 year old age group. In addition, it was unclear whether two participants had understood this question or not.

#### Relevance

Eight of the 20 participants asked (8/20, 40.0%) reported that their chest had felt ‘full of stuff’ during their cold, *‘not at all, I had no mucus today. I had a little mucus yesterday, but none today’* (0118-CC-M-74). Five participants (5/20, 25.0%) reported that they had not experienced this symptom during their cold, and seven participants did not explicitly make it clear whether or not they had experienced this symptom. This tended to be because participants had not made it clear whether or not they had understood the item and so symptom relevance could not be ascertained from participant responses.

#### Reword

Four of the five participants asked (4/5, 80.0%) stated that they would reword the item to improve overall understanding. These participants suggested rephrasing the item and/or to include a more accurate description of ‘stuff’. Table 61 provides some of the quotes from these participants.

Table 61. Participant suggestions for item ePRO 5d modifications

| **Suggestion** | **Quote** |
| --- | --- |
| Rephrase item and remove the word ‘full’ (n=2) | ***“How would you word that differently to make it a bit easier to understand?”*** *“Did your chest feel like it had all – a lot of stuff in it? Or did your chest just feel like it was normal, everything’s cool, everything.”* (0125-CC-F-8) |
| Replace ‘full of stuff’ with ‘clogged’ (n=1) | ***“How would you change that?”*** *“Uh, like how clogged it was.”* (0116) |
| Specify ‘stuff’ (n=1) | *“Uh, maybe instead of saying full of stuff, you just say full of boogers – or mucus or whatever.”* (0132-CC-M-16) |

#### Parent feedback on the ‘chest feels full’ items

Two parents reported that their child had not understood the phrase ‘stuffed up’ (0131-CC-M-6-P & 0112-CC-M-6-P), six parents stated that their child had difficulty understanding the term ‘clogged up’ and one parent reported that her child did not understand the term ‘mucus’ (0125-CC-F-8-P). Two parents reported that their children had not understood the phrase ‘clear chest’ and suggested using the term ‘empty’ instead (0131-CC-M-6-P & 0112-CC-M-6-P). Additionally, two parents stated that their children had difficulty understanding the term ‘full of stuff’ and suggested that the items should include a description of the word ‘stuff’ (0125-CC-F-8-P & 0120-CC-F-8-P). Another parent suggested including an explanation of ‘stuff’ in parentheses (0131-CC-M-6-P).

## Concept: Difficulty clearing mucus

Four items assessing the concept of ‘difficulty clearing mucus’ were debriefed with participants. Three items were tested on paper (Figure 38, Figure 39 and Figure 40) and one item was programmed onto the ePRO diary for testing in round two (Figure 41). The items assessing clearing the chest and throat were both fairly well understood and relevant items, with participants able to differentiate between the two symptoms. However, some of the younger children (6-8 year olds especially) were unclear what mucus/goo/gunk referred to.

### Paper6a: How hard was it to clear your throat?


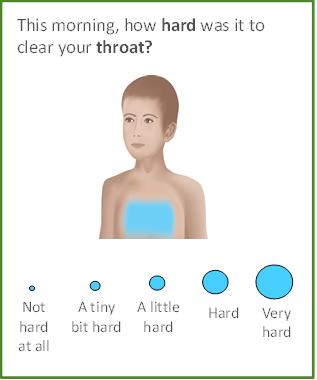


Figure 38. Paper: Item 6a

#### Understanding

The majority of the participants (46/49, 93.9%) in the sample were asked about their understanding of this item. Of these, 36 participants (36/46, 78.3%) demonstrated a good understanding of this item, *‘like when – sometimes when you have mucus, you try to clear your throat’* (0109-RC-F-10). Four of the participants asked (4/46, 8.7%) demonstrated that they had difficulty understanding the item. One participant thought it meant throat *‘pain’* (0122-RC-F-11), and one participant defined the question as *‘cleaning with water’* (0213-RC-M-6). The remaining two participants stated that they were not sure what the item meant. Two of these participants were in the 6-8 year old age group, and the remaining participant was in the 9-11 year old age group. Six participants had not made it clear whether or not they had understood the item.

#### Relevance

Twenty-three of the 33 participants asked (23/33, 69.7%) stated that this item related to a symptom that they had experienced during their cold, *‘I would choose, um, a little bit hard, because today it was a little bit hard to clear my throat in – while I was in class’* (0102-CC-F-11). Conversely, six participants (6/33, 18.2%) stated that that it was not difficult to clear their throat during their cold and reported that this was not a relevant item. It was not clear whether this symptom was relevant for four participants.

#### Reword

Only two of the ten (2/10, 20.0%) participants asked recommended rewording this item. One participant suggested changing the item to *‘this morning, how hard was it to – breathe, probably, so – so that – so that no mucus was in your throat’* (0119-CC-M-11). The other participant suggested changing the word ‘throat’ as the mucus may not be restricted to your throat, *‘because you could have like, um, post nasal drip or whatever’* (0214-CC-M-58). The remaining eight participants stated that they would not make any changes to the wording of the item.

### Paper6b: How hard was it to blow your nose?


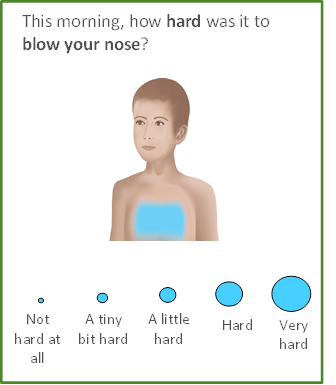


Figure 39. Paper: Item 6b

#### Understanding

Forty-three of the 49 participants (43/49, 87.8%) were asked about their understanding of this item. Forty of these 43 participants (40/43, 93.0%) appeared to understand this item, ‘*because there’s like a lot of snot in there, like so you blow it out, so it’s kind of easy’* (0129-CC-M-11). Only one eight year old had not understood this item, and interpreted the item as how much pain did blowing his nose cause, *‘when you blew your nose, did it hurt?’* (0105-CC-M-8) The remaining two participants did not make it clear during the interview whether they had understood the item or not.

#### Relevance

Twenty-three of the 38 participants asked (23/38, 60.5%) reported that they had experienced difficulty blowing their nose during their cold, *“Yeah, this week, it was kind of hard to blow my nose”* (0209-CC-F-16). Fifteen participants (15/23, 65.2%) said they had not experienced this symptom.

#### Reword

Three of the 11 participants asked (3/11, 27.34%) reported that they would reword the item. All three participants stated that it would be helpful to further clarify the item. Table 62 provides a quote from one of these participants.

Table 62. Participant suggestions for item Paper 6b modifications

| **Suggestion** | **Quote** |
| --- | --- |
| Further clarify item (n=3) | *“So this is a very ambiguous question – because I know it’s going to hurt. It’s a pain in the neck. How hard was it to blow my nose? Not hard. It’s simply take a tissue. Is – what do you mean by this?”* (0105-CC-M-58) |

### Paper 8a: How hard was it to cough up mucus (goo) from your chest?


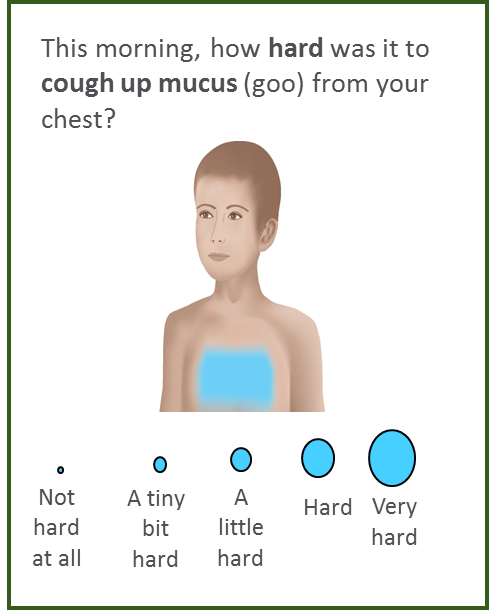


Figure 40. Paper: Item 8a

This item was only tested in round one as, following interim analysis, the word ‘goo’ was replaced with the word ‘gunk’. The reworded item was programmed onto the ePRO diary for testing in the second round of interviews. As a result, this item with the term ‘goo’ was debriefed with the 26 participants in round one and the item with the term ‘gunk’ was debriefed with the 23 participants in round two.

#### Understanding

Twenty of the 22 participants asked (20/22, 90.9%) demonstrated a good understanding of this item, *“um, it’s green or white, and it’s very sticky”* (0202-RC-F-9). One of the participants (1/22, 4.5%) had difficulty understanding this item and replied to the question by stating that *‘goo means like something is really bad in your chest, like – like bad food and like dirt, sometimes, um, sand’* (0120-CC-F-8). It was unclear whether the remaining participant had understood this item or not.

#### Relevance

Seventeen of the 21 participants asked (17/21, 81.0%) reported that they had experienced this symptom at some point during their cold, *‘Um, not hard at all. But when I had my cold, sometimes it would be hard’* (0109-RC-F-10). Four participants (4/21, 19.0%) reported that they had not experienced ‘coughing up mucus’ during their cold.

#### Reword

Of the 14 participants asked about this item, six participants (6/14, 42.9%) reported that they would modify the wording of the item. Of these participants, common suggestions were to replace terms such as ‘goo’ (as was done) and ‘mucus’ and/or to rephrase the item. Table 63 provides some of the quotes from these participants.

Table 63. Participant suggestions for item Paper8a modifications

| **Suggestion** | **Quote** |
| --- | --- |
| Remove the word ‘goo’ (n=1) | *“Leave out the word gook – hoo – whatever it is – everybody knows what mucus is.”* (0103-CC-F-70) |
| Replace ‘mucus’ with ‘congestion’ (n=1) | *“Uh, I would go with something more prettier – to say like congestion.”* (0108-CC-M-51) |
| Focus on nasal mucus (n=1) | *“This morning, how hard was it to cough up mucus, goo, from your – your nose?”* (0113-CC-M-9) |
| Replace ‘mucus/goo’ with ‘phlegm/snot’ (n=1) | *“How hard was it to clear your throat – uh, to clear your lungs – and I would use phlegm or snot.”* (0203-CC-F-50) |
| Rephrase item (n=1) | *“Um, maybe if I had to rephrase it, I could say how much did you – how much mucus did you cough up?”* (0208-RC-M-13) |
| Change to ‘clear your throat’ (n=1) | *“Um, yeah, I’d say the clear your throat one would be better.”* (0211-RC-M-17) |

### ePRO8b: How hard was it to cough up mucus (gunk) from your chest?

Figure 41. ePRO: Item 8b

This item was added following the interim analysis to determine whether participants were more familiar with the term ‘gunk’ or the tem ‘goo’, used in item Paper8a. Therefore, only the 23 participants in the second round of interviews were debriefed on this item.

#### Understanding

All 23 participants were asked about their understanding of this item. Of the 23 participants asked in the second round of interviews, only 13 participants (13/23, 56.5%) appeared to understand this item, *‘if it was easy or hard to cough up phlegm from your throat or chest’* (0127-CC-F-11). Five of the participants (5/23, 21.7%) did not understand the item. One participant thought that the term ‘gunk’ related to *‘junk’* and *‘sand’* (0120-CC-F-8), and the remaining four participants admitted they were not sure about the meaning of the item. All of the participants who had difficulty understanding this item were in the 6-8 year old age group. Five participants did not make it clear whether or not they had understood this item.

#### Relevance

Eleven of the 20 participants asked (11/20, 55.0%) reported that they had experienced this symptom during their cold. For instance, when asked to explain why one participant selected the option ‘very hard’, she responded *‘Um because when I coughed it hurt my chest – but once I started coughing a lot, my chest got worse, but the mucus inside was coming out’* (0216-CC-F-9). Conversely, six participants (6/20, 30.0%) reported that coughing up mucus was not a relevant symptom of their cold and it was unclear whether this was relevant for two participants.

#### Reword

Three of the seven participants asked (3/7, 42.9%) stated that they would reword the item. Participants typically suggested modifying and/or further clarifying the term ‘gunk’. Table 64 provides some of the quotes from participants.

Table 64. Participant suggestions for item ePRO8b modifications

| **Suggestion** | **Quote** |
| --- | --- |
| Replace ‘gunk’ with ‘spit’ (n=1) | ***“Would you ever use the word gunk?”*** *“Um, only for my ears but not – for this – I’d probably say the spit.”* (0129-CC-M-11) |
| Replace ‘gunk’ with alternative term (n=1) | *“Uh, so I wouldn’t use gunk. I’m not sure if it’s a very good word. I would use like snot or something different.”* (0132-CC-M-16) |
| Clarify item (n=1) | *“This is kind of ambiguous – it tells me that OK, I’m coughing up mucus, so how – how – how hard was it to do that, like difficult was it to do that?”* (0214-CC-M-58) |

#### Parent feedback of ‘difficulty clearing mucus’ items

In general, parents found these items to be well understood by their children. Two parents reported that their child had difficulty understanding the word mucus: *‘I don’t think he understand what mucus is yet’* (0120-CC-F-8-P).

## Concept: Noise when breathing

Three items assessing the concept of ‘noise when breathing’ were debriefed with the participants; one item on the ePRO diary (Figure 42) and two items on paper (Figure 43 and Figure 44). One item assessed a rattling noise, another assessed a sharp noise and the final item assessed wheezing. Although all three items were fairly well understood by participants, relevance of the items was poor, particularly for the items assessing a rattling and sharp noise.

### ePRO8: How much did you wheeze (make a noise) when you breathed?


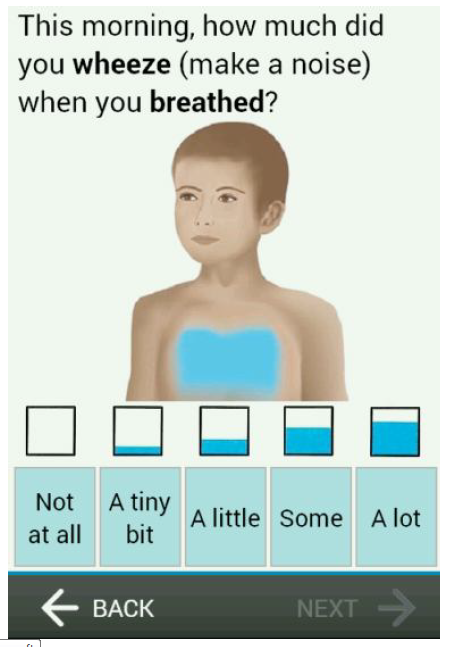


Figure 42. ePRO: Item 8

#### Understanding

Almost all of the participants were asked about their level of understanding of this question (48/49, 98.0%). Forty-one of the 48 participants asked (41/48, 85.4%) appeared to understand this item, *‘um, kind of like a high-pitched noise when you breathe’* (0208-RC-M-13). Six participants (6/48, 12.5%) had difficulty understanding this item. One participant referred to the noise as coming from the *‘nose’* (0122-RC-F-11), one participant thought the question meant the noise was coming from the *‘stomach’* (0213-RC-M-6), and another thought that it meant *‘water on the lungs’* (0203-CC-F-50). The remaining three participants admitted that they had not understood what this item meant. All six participants who had difficulty were in the 6-8 year old age group. For the remaining participant, level of understanding was unclear.

#### Relevance

Twenty-five of the 44 participants asked (25/44, 56.8%) reported that this was a relevant symptom for them, *‘I didn’t hear it as often as I did when I – when my cold was bad’* (0205-CC-M-14). Sixteen participants (16/44, 36.4%) said that they had not experienced this symptom during their cold and level of relevance was unclear for three participants.

#### Reword

Three of the 20 participants asked (3/20, 15.0%) suggested rewording the item. Both participants suggested that it would be beneficial to remove the term ‘wheeze’. Table 65 provides some quotes from these participants.

Table 65. Participant suggestions for item ePRO 8 modifications

| **Suggestion** | **Quote** |
| --- | --- |
| Replace ‘wheeze’ with ‘whistle’ (n=2) | *“I think you should change the question to like hearing a little whistle coming – like whistle when you’re breathing.”* (0102-CC-F-11) |
| Remove the term ‘wheeze’ (n=1) | *“I Don’t consider wheezing with breathing. I just consider it, like, you know, um, you know, was it hard to breathe this morning.”* (0104-CC-F-50) |

### Paper7a: How much did your chest make a rattling noise when you breathed?


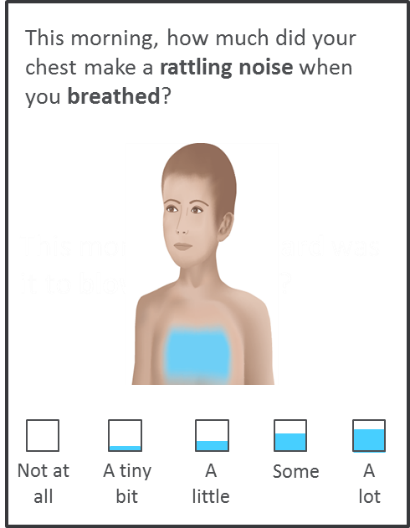


Figure 43. Paper: Item 7a

#### Understanding

Almost all of the participants (48/49, 98.0%) were debriefed on this alternative version of the noise item. Of these 48 participants asked, 35 participants (35/48, 72.9%) demonstrated a good understanding of this item, *‘rattling noise is pretty much saying it’s another word for wheezing’* (0205-CC-M-14).

Twelve participants had difficulty understanding this item and required further clarification of the term ‘rattling’. Two of these participants associated the rattling noise with coming from their stomach rather than their chest (0122-RC-F-11 & 0129-CC-M-11). The remaining ten participants did not understand either the entire item or specifically the term ‘rattling noise’. Despite several younger participants not understanding this item, several of the older participants also failed to understand this item, suggesting that collectively it was a poorly understood item. The participants who had difficulty understanding this item were of a variety of ages and didn’t fall into one age category. Four of these participants were in the 6-8 years age group, three participants were in the 9-11 years age group, three participants were in the 12-17 years age group and two participants were in the adult age category. In addition, the one remaining participant did not made it clear whether or not he had understood this symptom.

#### Relevance

In addition to the relatively poor understanding of this item, very few participants reported that item was relevant to their cold. In total, only 12 of the 39 participants asked (12/39, 30.8%) reported that they had noticed a ‘rattling’ noise when breathing during their cold. For example, one participant described this as a ‘whistling noise’ and stated that she *‘had it towards the beginning and middle of my cold – it made me sound like I was blowing a whistle’* (0102-CC-F-11). Twenty-six participants (26/39, 66.7%) reported that they had not heard a rattling noise when breathing, and it was unclear whether one participant had experienced this or not.

#### Reword

Eight of the 11 participants asked (8/11, 72.7%) suggested a modification to the item to improve overall understanding. The majority of these participants suggested replacing and/or removing the specific term to describe the breathing noise. Table 66 describes the different suggestions made by several participants.

Table 66. Participant suggestions for item Paper7a modifications

| **Suggestion** | **Quote** |
| --- | --- |
| Remove ‘rattling noise’ (n=4) | *“I have rattlesnakes in my chest? That’s my first thought when I read that. I think that has to be gotten rid of – plus it doesn’t make sense.”* (0211-RC-M-17) |
| Replace ‘rattling noise’ with ‘wheezing’ (n=1) | *“I’d probably use wheezing.”* (0103-CC-F-70) |
| Rephrase and remove specific terms to describe the noise (n=1) | *“You know, was it – did you experience any noise when you breathed or something.”* (0104-CC-F-50) |
| Rephrase item (n=1) | *“7a is awkwardly phrased.”* (0105-CC-M-58) |
| Alternative suggestion (n=1) | *“Um this afternoon, how much did your chest make a like, um – a like noise that wouldn’t be like – like you could explain. I don’t know – probably like sort of like a bunch of like stomps or like thumping – like when you were breathing.”* (0210-CC-F-14) |

### Paper7b: How much have you noticed a sharp noise when you breathed in or out?


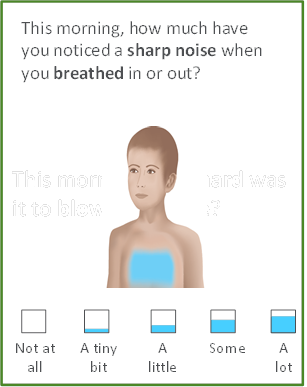


Figure 44. Paper: Item 7b

#### Understanding

Thirty of the 44 participants asked (30/44, 68.2%) demonstrated a good level of understanding of this item, *‘I understand this one as asking me if I had really sharp whistling sounds when I’m breathing’* (0115-CC-F-11). However, a large proportion of the participants (14/44, 31.8%) failed to understand the item and were subsequently not able to respond. Twelve of these participants reported that they did not understand the term ‘sharp noise’. One participant interpreted a sharp noise to mean a ‘hard noise’ (0128-CC-M-7), and one participant thought it meant *‘sharp like a pen’* (0107-RC-M-8). The participants who did not understand this item were of a variety of ages; seven participants were in the 6-8 years age group, one participant was in the 9-11 years age group, another in the 12-17 years age group and one participant was in the adult age category.

#### Relevance

Of the 35 participants asked, only eight participants (8/35, 22.9%) stated that they had heard a ‘sharp noise’ when breathing during their cold, *‘um, yes, I had it towards the beginning and the middle’* (0102-CC-F-11) and twenty-six participants (26/35, 74.3%) reported that they had not experienced hearing any sharp noises during their cold. One participant did not make it clear whether he had experienced a sharp noise.

#### Reword

Thirteen of the 15 participants asked (13/15, 86.7%) reported that they would reword this item, which suggests that this item needed to be amended. The majority of participants suggested asking more generically about a noise rather than using a specific term to describe that noise. Table 67 outlines the suggestions made by these participants.

Table 67. Participant suggestions for item Paper 7b modifications

| **Suggestion** | **Quote** |
| --- | --- |
| Ask more generically about noise – do not specify term to describe the noise (n=5) | *“Um, I think just put like a noise – yeah make a noise.”* (0129-CC-M-11) |
| Reword (n=4) | *“Just like when you br – I don’t know how I’d describe it – it’s kind of a tricky question, I guess.”* (0209-CC-F-16) |
| Remove ‘sharp’ – add term that refers to water (n=1) | *“Sharp isn’t actually – it described – uh, I don’t know how it can describe a noise – I would use like gurgle – um, even bubbly you know – just something that would relate to – the sound of water.”* (0203-CC-F-50) |
| Clarify what a ‘sharp noise’ is (n=1) | *“I’m not sure what it’s asking – I guess have anybody describe what the noise is if they breathed out or in.”* (0204-CC-F-45) |
| Replace ‘sharp noise’ with ‘short noise’ (n=1) | *“Um, maybe rephrase sharp noise – maybe something like a short noise if that’s actually a correct – synonym.”* (0208-RC-M-13) |
| Replace ‘sharp’ with ‘wheeze’ (n=1) | *“I’d choose the wheezing, because it covers – especially how it was worded where it‘s saying – what did it say? – and then in parentheses said or n – make noise or something.”* (0211-RC-M-17) |

#### Parent feedback of ‘noise when breathing’ items

Six parents stated that their children did not understand the term ‘wheezing’, *‘he no way would understand wheezing’* (0120-CC-F-8-P). Two parents stated that their child did not understand the term ‘rattling noise’ (0206-CC-M-7-P & 0126-CC-F-7-P) and the remaining two parents reported that their children did not understand the term ‘sharp noise’ (0101-CC-F-8-P & 0131-CC-M-6-P). The majority of parents suggested that they would phrase the item as ‘making a noise’ rather than a more complex description, *“Uh, I’d probably say this afternoon, how much did you make a noise whenever you breathed?”* (0112-CC-M-6-P)

### Response options

All items had a five point response scale with a range of different verbal descriptors. The response option descriptions included: ‘not hard at all’ to ‘very hard’, ‘not tight at all’ to ‘very tight’, ‘not at all’ to ‘a lot’ and ‘not clear at all’ to ‘very clear’.

Participants were specifically asked to provide feedback on how the response options looked visually (e.g. the filled boxes and circles of increasing sizes). Overall, participants found the response options easy to understand and did not experience any difficulties, *‘just by like you can see like the bigger ones are like it hurts more, so it’s just kind of like more visual’* (0114-RC-F-14)*.* Only one of the 49 participants (1/49, 2.0%) found the response options confusing, *‘But the wording – like on the bottom, like under the blue – it says like not hard at all but like – and like easy and that – but shouldn’t it say like normal?’* (0210-CC-F-14) Five participants suggested making changes to the response options, which have been outlined in Table 68.

Table 68. Suggested changes to response options

| **Suggestion** | **Quote** |
| --- | --- |
| Narrow the options | *“I would even say they could narrow the choices down a bit.”* (0108-CC-M-51) |
| Scale of 1-10 | *“I would like to see something different – a scan of one to 10, how was like – how was your chest feeling.”* (0122-RC-F-11) |
| Square fillings | *“Change the squares all the way up.”* (0131-CC-M-6) |
| Faces | *“Um – like maybe, faces – like if it was OK, then smiley face or really bad and it’s like crying or crying”* (0124-CC-F-11) |
| Consistently use circles or boxes | *“But you need to go with one of the – you need to – I don’t know if you should vary it or –“* (0105-CC-M-58) |

#### Filled boxes response options

Seventeen participants reported that they liked the filled boxes response options, *‘the boxes I like really much’* (0120-CC-F-8). Only two participants mentioned that they did not like the box response scales. One participant reported to find the boxes *‘confusing’* (0124-CC-F-11), and the remaining participant did not like the fact that the boxes were never totally full at the higher end of the response scale (0131-CC-M-6). Despite two participants not liking the squares, all participants understood the response options.

Fourteen participants found the boxes helpful while four participants did not need the boxes, *‘Mm, I don’t think they make a difference’* (0127-CC-F-11). Eight participants reported that they preferred answering using the box response scale rather than the circle response scale, *‘the squares would’ve been easier, because it shows – like the full thing can be this and it shows how big it can get’* (0111-CC-M-12). Of those participants who preferred the filled boxes response options, there were no observable differences as reported by age; 6-8 years old (n=3), 9-11 years old (n=3), 12-17 years old (n=1) and adults (n=1).

#### Circle response options

Twenty-seven participants stated that they liked the circles of increasing sizes response options, *‘I thought the circles were a good choice of like saying – like this much or like – how big of a portion of how you’re feeling’* (0102-CC-F-11). Three participants mentioned an aspect of the response scale that they did not like. Two of these participants found each of the circles to be too similar in size (0210-CC-F-14 & 0211-RC-M-17), and the remaining participant did not like that each of the circles was filled in with colour (0128-CC-M-7), *‘the not hard at all and – and tiny bit hard appear to be the same size up here – so that’s kind of useless’* (0211-RC-M-17). All participants understand the circular response options.

Sixteen participants reported that the circle response options were helpful, *‘well, um, it helped me answer the questions by, um – by telling me if it’s no, no, maybe a little, or whatever’* (0120-CC-F-8). Five participants stated that they did not find the circles helpful, *‘um, they didn’t really mean that much to me, I just looked at the words usually’* (0208-RC-M-13). Twelve of the 49 participants (12/49, 24.5%) stated that they preferred answering the questions using the circles rather than the boxes, *‘I’d rather do circles – circles make more sense, because small, medium, large’* (0113-CC-M-9). Of those 12 participants who preferred the circle response options, there were no observable differences as reported by age; 6-8 years old (n=3), 9-11 years old (n=4), and 12-17 years old (n=3).

#### Illustrations

Participants generally understood and liked the illustrations (drawings of a child experiencing the symptom) which accompanied each item and found them helpful. Four participants suggested making the illustrations ‘animated’ and/or ‘interactive’, *‘Did your chest feel full of mucus, the goo that – OK, so basically for this one, you could have – that blue thing with a little line coloured in with some liquid’* (0105-CC-M-58). Two participants explained that they would find it more beneficial to highlight a different area of each picture to demonstrate the area of the body that the participant should focus on.

### Recall period

For the morning diary, the recall period was ‘this morning’ and for the afternoon diary, the recall period was ‘this afternoon’. The appropriateness and level of understanding of the recall period was assessed in the CD interview. At the beginning of the interview, participants were asked general questions to assess how easy or difficult it was to recall their symptoms from the morning, the afternoon or the previous night. During the ‘think aloud’ exercise, participants were asked to provide feedback on the recall period they were using for specific items.

During the general questions, thirty-five participants (35/49, 71.4%) were asked to provide feedback on the afternoon recall period. Of these, 32 participants (32/35, 91.4%) stated that they found the recall ‘easy’ and ‘simple’, *‘um, the afternoon was clearer for me, just because – I hadn’t just woken up – I had gone through most of my day’* (0218-CC-M-16). Three of the 35 participants (3/35, 8.6%) reported some difficulty recalling their symptoms over the afternoon period as it was a ‘long’ time period to think back over (0104-CC-F-50, 0206-CC-M-7, 0217-RC-F-7).

Twenty-three participants (23/49, 46.9%) were asked to provide feedback on the morning recall period. Twenty-one of the 23 participants (21/23, 91.3%) stated it was ‘easy’ to remember any symptoms that they had experienced that morning, *‘it was easy to do, because I remembered, you know. I did it early in the morning, as soon as I got up’* (0103-CC-F-70). The remaining two participants (2/23, 8.7%) reported that they found it challenging to recall their symptoms from the morning. One participant reported that he found it more difficult due to reduced concentration levels during the morning (0218-CC-M-16), and the remaining 6 year old participant stated she could not remember, while her parent added that *‘at her age they don’t have a sense of time’* (0117-RC-F-6).

When asked whether participants found it easy or difficult to recall their symptoms over the previous night, 28 of the 30 participants asked (28/30, 93.3%) stated that they found it ‘easy to remember’, *‘uh, very easy, because if it didn’t feel good during the night, I would be waking up coughing and wheezing or whatever, so very easy for me’* (0105-CC-M-58). The remaining two participants found it difficult to recall their symptoms from the previous night but neither elaborated on this (0116-CC-M-10 & 0117-RC-F-6).

Following the general discussion, participants were asked more specifically about the recall periods for each of the items during the ‘think aloud’ task. Typical questions to assess the comprehension of the recall period included ‘when were you thinking about when answering this question?’ and/or ‘was it easy or difficult to remember?’ Fifteen of the 49 participants (15/49, 30.6%) either misinterpreted or had difficulty with the recall period for one or more of the items. Five participants found it ‘difficult’ to remember back over the recall period for the symptom being assessed, *‘you answer the question first thing in the morning – and, um, it’s, like, overnight, you know, you’re sleeping’* (0104-CC-F-50). Three participants reported that they were thinking over the ‘whole day’ when answering an item rather than more specifically the ‘morning’ or ‘afternoon’ (0124-CC-F-11, 0210-CC-F-14 & 0213-RC-M-6). Three participants reported to be answering an item based on a specific moment in time, for example, ’11.45pm’ (0106-CC-F-53, 0125-CC-F-8, 0216-CC-F-9). However, this may simply be an artefact of the artificial nature of the CD interview; it does not necessarily reflect the period they would have recalled over during a study. Two participants reported that they were thinking about the first day of their cold when their symptoms were slightly worse when answering one or more items (0119-CC-M-11 & 0207-CC-M-26). Two participants were referring to the ‘morning’ when answering an afternoon item and vice versa (0210-CC-F-14 & 0121-CC-M-12). There did not appear to be any observable differences between the age groups of participants who reported difficulties with the recall period: three in the 6-8 year old year old age group, four in the 9-11 year old age group, five in the 12-17 year old age group and the remaining three in the adult age category. For those participants that used an inappropriate recall period for at least one item, they used the correct recall period for the majority of items.

In summary, while there were some examples of incorrect recall periods being used, in general the recall periods were well understood and used correctly. For example, when one participant was asked at what time he was thinking back to when answering a morning item, he responded, *“Maybe like 6:00 to like 8:00 – in the morning.”* (0208-RC-M-13). A further participant stated, *‘I was thinking about, um, how I was feeling during that afternoon and how I was feeling right now’* when she answered an afternoon item (0124-CC-F-11).

## Rationale for retaining or deleting items

Following the final results presentation, the research team discussed all the items included in both Round 1 and Round 2 and came to a consensus agreement about the items to retain and test in the subsequent psychometric validation study. A detailed rationale for each of these decisions is provided in the following sections.

### Difficulty breathing

During the CE interviews, difficulty breathing was identified as the most frequently experienced symptom. Two items assessing difficulty breathing were cognitively debriefed with participants, one on paper and one on the ePRO diary. The wording of the two items was identical except that the paper item included ‘because of your cold’ at the end. Both items were well understood and equally relevant to participants. It was agreed to retain the item ‘how hard was it to breathe air deep into your chest?’ and remove the additional wording of ‘because of your cold’ to reduce the number of words in the item and make the item as simple as possible for the children to read.

### Chest tightness

Although a more complex symptom, chest tightness was reported by over half of the participants (29/49, 59.2%) during the CE interviews and was well understood by most participants during the CD interviews. However, debriefing results from parents and children suggested some 6-11 year olds and some adolescents had difficulty understanding ‘tight’.

Although it appeared that item Paper2 (‘how tight did your chest feel?’) was less relevant than item ePRO2 (‘how tight did your chest feel because of your cold?’), the paper item was asked to fewer participants. For the same rationale as the difficulty breathing items, it was agreed to retain the item ‘how tight did your chest feel?’ and remove the additional wording of ‘because of your cold’ to reduce the number of words in the item and make the item as simple as possible for the children to read.

### Chest pain

Prior to the interviews chest pain was not understood to be a relevant symptom associated with chest congestion and therefore was not included as a topic of interest in the original interview guide. However, during round one interviews almost half of the sample spontaneously discussed chest pain as a symptom of their cold and so this symptom was added to the interview guide as a topic for discussion in the round two interviews.

Two items assessing chest pain were debriefed with participants. Both items assessed chest pain in relation to another chest symptom (i.e. chest hurt due to coughing and chest hurt due to being stuffed up). Chest pain due to coughing was well understood and relevant to the majority of participants whereas chest pain due to being stuffed up was poorly understood across the age groups mainly due to difficulty understanding ‘stuffed up’. Consequently, the item ‘how much has your chest hurt when you’ve coughed’ was retained and the item ‘how much has your chest hurt due to being stuffed up’ was deleted.

### Chest feels heavy

During CE, chest feels heavy (or chest heaviness) was reported by over half of the participants as a symptom they had experienced as part of their cold. Although only eight participants (8/49, 16.3%) reported this symptom spontaneously during CE, 35 participants (35/49, 71.4%) reported that this was a relevant symptom of their cold during the CD interviews.

Two items assessing chest heaviness were debriefed with participants. One item asked directly about the level of heaviness experienced in the chest and the other asked about the amount of time that the chest felt heavy. Both items performed similarly in CD, so it was agreed to retain the item ‘how heavy did your chest feel?’ as it followed the same format as the chest tightness item ‘how tight did your chest feel’. Additionally, this item asked directly about the participant experience of chest heaviness rather than the duration of the chest heaviness (i.e. amount of time) and it was agreed that this would be easier for the children to understand.

Of note, like chest tightness, the feedback from parents and children did suggest that the chest feeling heavy was a difficult concept for some of the younger children to understand. It is recommended that parents are trained to help the children understand the item during future studies.

### Chest feels full

The concept of ‘chest feels full’ was explored through the sub-concepts of ‘stuff in chest’ and ‘stuffed up or clogged up’. Although fairly few participants reported the concepts spontaneously, over half of the participants reported experiencing these symptoms when probed, suggesting that participants did experience this symptom.

During CD, several items measuring the same symptom but with alternative wording were tested to establish the most understandable and relevant item. The newly added item ePRO5d (‘how full of stuff did your chest feel’) was misunderstood by almost a third of the participants asked and was therefore deleted. The item ePRO5 (‘how much did your chest feel full of mucus [the goo that comes out of your nose/goo]’) performed equally well with the term ‘goo’ rather than ‘the goo that comes out of your nose’ and it was agreed that the item with the fewer words would be retained as it is easier for children to read and understand.

Both Paper5b (‘how much did you feel clogged up in your chest’) and Paper5c (‘how clogged up did your chest feel’) measured the symptom ‘clogged up’. The items performed similarly in terms of level of understanding and relevance. Both Paper5a (‘how much did you feel stuffed up in your chest’) and ePRO6 (‘how stuffed up did your chest feel?’) measured the symptom ‘stuffed up’. These items also performed similarly in terms of level of understanding and relevance. It was agreed that, in both cases, the items with the simpler wording would be retained. Of note, although understanding was high, more patients and parents suggested rewording the clogged up items suggesting the wording is perhaps less natural than others tested. Therefore, while one clogged up item was retained it is likely it will not perform as strongly and it could be a candidate for deletion following the observational study.

Although item Paper6c (‘how clear did your chest feel’) was fairly well understood by participants, this was the only item where the response scale was reversed (e.g. participants reported on how well they felt rather than how unwell they felt). Due to the potential errors associated with only one item having a reversed scale, it was agreed that this item would be deleted.

### Difficulty clearing mucus

The symptom of ‘difficulty clearing mucus’ (along with ‘chest pain’) was the most frequently reported spontaneous symptom and participants talked openly about their experience of this symptom.

Difficulty clearing your chest (item ePRO7) and difficulty clearing your throat (item Paper6a) were both fairly well understood and relevant items and participants were able to differentiate between the two symptoms. Although some of the younger children (6-8 year olds especially) were unclear what mucus/goo/gunk referred to. As difficulty clearing mucus is a key symptom of chest congestion and Guaifenesin is indicated to help loosen and thin mucus, it was agreed that both of these items should be retained to fully assess this concept.

The item assessing coughing up mucus (Paper8a/ePRO8b) was amended between rounds and the description changed from ‘goo’ to ‘gunk’. The item tested in round one, with the term ‘goo’ was better understood and relevant to more participants and therefore this item wording was retained. Although the majority of participants understood item ePRO6b, difficult blowing your nose was not considered adequately relevant to chest congestion and it was agreed that this item should be deleted.

### Noise when breathing

During the CE interviews, noise when breathing was the least frequently experienced symptom and was only mentioned spontaneously by four participants. Three items assessing noise when breathing were debriefed with participants. One item assessed a rattling noise, another assessed a sharp noise and the final item assessed wheezing. Although all three items were fairly well understood by participants, relevance of the items was poor, specifically for the items assessing a rattling and sharp noise. Participants reported that the item assessing wheezing was the most relevant to the symptoms they experienced during their cold, however, following further discussion it was agreed that noise when breathing was not a concept of interest for the naturalistic study and it is not indicated as a target symptom for Guaifenesin. Consequently, all three items were deleted.
